# Whole genome sequencing and annotation of *Daedaleopsis sinensis*, a wood-decaying fungus significantly degrading lignocellulose

**DOI:** 10.3389/fbioe.2023.1325088

**Published:** 2024-01-16

**Authors:** Jin-Xin Ma, Hao Wang, Can Jin, Yi-Fan Ye, Lu-Xin Tang, Jing Si, Jie Song

**Affiliations:** ^1^ Institute of Microbiology, School of Ecology and Nature Conservation, Beijing Forestry University, Beijing, China; ^2^ Department of Horticulture and Food, Guangdong Eco-Engineering Polytechnic, Guangzhou, China

**Keywords:** wood-decaying fungi, whole genome, lignocellulose degradation, secondary metabolism, gene annotation

## Abstract

*Daedaleopsis sinensis* is a fungus that grows on wood and secretes a series of enzymes to degrade cellulose, hemicellulose, and lignin and cause wood rot decay. Wood-decaying fungi have ecological, economic, edible, and medicinal functions. Furthermore, the use of microorganisms to biodegrade lignocellulose has high application value. Genome sequencing has allowed microorganisms to be analyzed from the aspects of genome characteristics, genome function annotation, metabolic pathways, and comparative genomics. Subsequently, the relevant information regarding lignocellulosic degradation has been mined by bioinformatics. Here, we sequenced and analyzed the genome of *D. sinensis* for the first time. A 51.67-Mb genome sequence was assembled to 24 contigs, which led to the prediction of 12,153 protein-coding genes. Kyoto Encyclopedia of Genes and Genomes database analysis of the *D. sinensis* data revealed that 3,831 genes are involved in almost 120 metabolic pathways. According to the Carbohydrate-Active Enzyme database, 481 enzymes are found in *D. sinensis*, of which glycoside hydrolases are the most abundant. The genome sequence of *D. sinensis* provides insights into its lignocellulosic degradation and subsequent applications.

## 1 Introduction

Most wood-decaying fungi are classed as basidiomycetes. Wood-decaying fungi can spread in wood through mycelia and secrete a series of enzymes to decompose the intertwined long-chain macromolecules cellulose, hemicellulose, and lignin in wood tissues (Si et al., 2013b; Zheng et al., 2016; Zheng et al., 2017; Ma et al., 2018; Si et al., 2021a; Si et al., 2021b). Simultaneously, the fungi can use the resulting starch and sugars as nourishment to meet their own growth and reproduction. Wood-decaying fungi have substantial ecological, economic, edible, and medicinal value. First, some small molecular substances degraded by wood-decaying fungi can be used for industrial production, such as bioethanol production. In addition, the enzyme system produced by wood-decaying fungi in the degradation process has a variety of applications; For example, laccase can be used to degrade pollutants, but also can synthesize antibiotics, amino acids, and other large molecular compounds. Second, many wood-decaying fungi are edible and can be used in medicine. Third, wood-decaying fungi are a vital part of the forest ecosystem and play an important role in degradation and reduction (Lonsdale et al., 2008; Krah et al., 2018; Li et al., 2022). The dead branches, fallen leaves, and decayed wood that are degraded by fungi can be returned to nature, thus participating in the material circulation and energy flow of the whole ecosystem, promoting metabolism, and maintaining dynamic balance. The wood residues degraded by brown rot fungi can remain in the soil for more than 3,000 years, which can increase soil ventilation and water retention. This process promotes the formation of ectomycorrhiza and the nitrogen-fixing ability of non-symbiotic microorganisms, as well as improving soil temperature, reducing soil pH, and increasing cation exchange in nutrients, all of which are crucial for seed germination and seedling development (Larsen et al., 1979; Larsen et al., 1982). In addition, wood-decaying fungi are closely related to other organisms that serve important ecological functions. For example, many insects and birds acquire the nutrients they need for their growth and development from wood-decaying fungi, and some of these insects also spread in spores. Therefore, wood-decaying fungi are an indispensable part of the forest ecosystem, and protecting and utilizing wood-decaying fungi is essential to protect the ecosystem and maintain ecosystem health.

Lignocellulose is composed of cellulose, hemicellulose, and lignin intertwined to form a dense network structure. Cellulose is a linear chain polysaccharide of glucose units connected by *β*-1,4 glucoside bonds (Liu et al., 2019; Kargar et al., 2021), and part of the linear long chain is connected by hydrogen bonds and van der Waals forces, showing a clear X-ray diffraction pattern that is called the crystallization zone. The other part of the molecular chain arrangement is an unordered, irregular, and relatively relaxed region termed the amorphous region (Zugenmaier, 2021). Cellulose is the most abundant natural polymer in the world. It is cheap, degradable, and environmentally friendly. Hemicellulose is the second type of polysaccharide in the plant fiber raw material contents. Hemicellulose is a low-molecular-weight branched polymer that is composed of two or more monosaccharides, mainly connected by *β*-1,4 glycosidic bonds, and has a high degree of branching and a certain degree of substitution (Sun et al., 2022; Rao et al., 2023). Lignin is a complex phenolic polymer that is renewable in nature and exists in the wood cell wall. The complex structure of lignin includes a rich variety of reactive groups, meaning this polymer can undergo various chemical reactions. Lignin can be converted into energy, chemicals, and functional materials, partially replacing fossil fuel-based products; hence, lignin is the third largest renewable biomass energy source after cellulose and hemicellulose (Liu et al., 2019; Ralph et al., 2019; Yang et al., 2020). The structural units of lignin are connected by ether and carbon-carbon bonds, forming a natural polymer with a three-dimensional structure (Sjöström, 1981; Linger et al., 2014; Sun et al., 2022). As the most abundant renewable resource on earth, lignocellulose is expected to replace fossil fuels as the main feedstock for biofuels. Prior to utilization, pretreatment is a critical step in the conversion of lignocellulose into fermentable sugars and biofuels (Wan and Li, 2012; Ponnusamy et al., 2019). Currently, white rot fungi are usually used as pretreated microorganisms, which can improve conversion efficiency through invading and destroying the protective barrier of lignocellulosic biomass feedstock with numerous advantages including high selectivity, low energy demand, nontoxic by-products, and sustainable development (Wan and Li, 2012). For pulping industry, the fungal pretreatment can significantly enhance paper strength, improve the dense energy input of mechanical pulping, and reduce the toxicity of pulping wastes (Kang et al., 2003; Rullifank et al., 2020). In conclusion, fungi play a very important role in the many industrial areas, and their genetic resources need to be further explored and developed.

*Daedaleopsis sinensis* is wood-decaying fungus that mainly infects the trunk of *Betula* and *Alnus* trees, is predominantly distributed in natural forests in Heilongjiang and Jilin, and causes white decay, which is common in nature (Dai, 2012). This species has coarse annual to biennial basidiocarps with large angular pores, pileate, and planar to triangular shapes (Núñez and Ryvarden, 2001; Dai and Penttilä, 2006; Li et al., 2016). Previously, several wood-decaying fungal strains with strong enzyme-secreting abilities were selected through guaiacol-containing solid plates and flask liquid cultivation, including *D. sinensis* used in the present study (Si et al., 2013a). Therefore, it was considered that this species has potential ability to degrade lignocellulosic materials.

Advances in DNA sequencing technologies have facilitated studies based on the genome sequences of macrofungi in addition to the continuing research on the metabolites themselves. *Ganoderma lingzhi* (Chen et al., 2012; Liu et al., 2012), *Taiwanofungus camphoratus* (Lu et al., 2014; Yang et al., 2018), *Hericium erinaceus* (Chen et al., 2017; Gong et al., 2020), *Auricularia heimuer* (Yuan et al., 2019), *Russula griseocarnosa* (Yu et al., 2020), and *Phanerochaete chrysosporium* (Martinez et al., 2004) have been used for whole genome sequencing. In this study, we sequenced the genome of *D. sinensis* and analyzed its functional annotation information to provide insights into secondary metabolism and carbohydrate metabolism.

## 2 Materials and methods

### 2.1 Strain culture and DNA isolation

The monokaryotic strain was isolated from a wild fruiting body collected from Changbai Mt., Antu County, Yanbian, Jilin Province, China. The mycelia of *D. sinensis* were harvested after growing on sterile cellophane covering potato dextrose agar (PDA) medium at 28°C for 10–14 days. The mycelia were collected, snap frozen in liquid nitrogen, and then stored at −80°C for further use. The enriched genomic DNA was extracted from the mycelia with a QIAGEN Genomic Kit and detected by agarose gel electrophoresis.

### 2.2 Genome sequencing and assembly

PacBio Sequel and MGISEQ2000 platforms were used for whole genome sequencing at Wuhan Nextomics Biosciences Co., Ltd. (Wuhan, China).

The quality of the DNA was examined using four methods: the DNA was inspected whether the appearance and shape of the sample contained foreign bodies; degradation and DNA fragment size of samples were detected by 0.75% agarose electrophoresis; DNA purity was measured by NanoDrop spectrophotometer; and precise quantification of the DNA was performed using a Qubit system.

After the samples passed the quality inspection, the genomic DNA was sheared with g-TUBEs (Covaris, United States) according to the size of the fragments built in the library, and magnetic beads were used to enrich and purify the targeted fragments of DNA. The fragmented DNA was then repaired for damage and terminal repair. Stem loop sequencing splices were connected at both ends of the DNA fragment, and exonuclease was used to remove the fragments that failed to connect. Next, BluePippin (Sage Science, United States) was used to screen the target fragments, and the library was purified. The library fragment size was then measured with an Agilent 2,100 Bioanalyzer (Agilent Technologies, United States). After construction of the library, DNA templates and enzyme complexes with a certain concentration and volume were transferred to the nanopore of the PacBio Sequel series sequencer for real-time single molecule sequencing.

Quality control was conducted on the raw sequencing data, and the low-quality areas and adapter sequences were removed to obtain high-quality DNA sequence data. Hifiasm software (parameter: −n5) was used for pure three-generation assembly to obtain the preliminary assembled genome sequence. Nextpolish software was used to calibrate the genome sequence four times with the second-generation data, and the final genome sequence was obtained.

### 2.3 Gene prediction and annotation

For genome repeat sequence prediction, GMATA version 2.2 (Wang and Wang, 2016) was used to analyze the simple sequence repeats in the genome. TRF version 4.07b (Benson, 1999) was used to analyze tandem repeats in the genome with default parameters. The interspersed repetitive sequences were predicted using RepeatMasker version open-4.0.9 (Bedell et al., 2000). Three methods were employed in gene structure prediction, namely, transcriptome prediction, homologous protein prediction and *de novo* prediction. Based on transcriptome sequence data, genome gene prediction was performed by PASA version 2.3.3 (Haas et al., 2003). Homologous protein prediction used GeMoMa version 1.6.1 (Keilwagen et al., 2016) to compare the corresponding protein information with the genome. In addition, *de novo* prediction was performed using Augustus version 3.3.1 (Stanke et al., 2008). Subsequently, the gene prediction results obtained by the above three methods were integrated by using EVidenceModeler version 1.1.1 (Haas et al., 2008). Genome integrity was assessed using Benchmarking Universal Single-Copy Orthologs (BUSCO) version 3.0.1 (Simão et al., 2015).

Gene functions were predicted with references to nine databases: Kyoto Encyclopedia of Genes and Genomes (KEGG) database (https://www.kegg.jp/) to understand the higher functions and utility of cells, organisms, and ecosystems at the level of molecular information and to reveal the hidden features in biological data (Kanehisa et al., 2017), Gene Ontology (GO) database (http://www.geneontology.org) to understand the living organisms in three pathways: molecular function, cellular component, and biological process (Ashburner et al., 2000); Eukaryotic Orthologous Group of Protein (KOG) database (https://www.creative-proteomics.com/services/kog-annotation-analysis-service.htm), a eukaryote-specific version of the Clusters of Orthologous Groups tool for identifying the ortholog and paralog proteins (Galperin et al., 2015); Non-Redundant Protein (NR) database (https://www.ncbi.nlm.nih.gov/protein/), a non-redundant protein library for protein function annotation; Fungal Cytochrome P450 (CYP) database (http://p450.riceblast.snu.ac.kr/cyp.php) and CYP Engineering database (http://www.cyped.uni-stuttgart.de) to classify and analyze the CYP monooxygenase family for a better understanding of their biochemical characteristics and sequence-structure-function relationships (Sirim et al., 2009; Moktali et al., 2012); Swiss-Prot database (https://www.uniprot.org/), a protein sequence library designed to provide the high levels of annotation, minimal redundancy, and integration with other databases (Stanke and Waack, 2004; Stanke et al., 2006); Pfam database (http://pfam.xfam.org/), an aggregation of protein families including comparative sequences, species information, and hidden Markov models corresponding to special gene families (Mistry et al., 2021); Carbohydrate-Active Enzyme (CAZyme) database (http://www.cazy.org/) (Drula et al., 2022), a special database dedicated to presentation and analysis of genomic, structural, and biochemical information on CAZymes. CAZyme database classifies CAZymes into different protein families based on the similarity of amino acid sequences in the protein domain, which covers related enzymes required for lignocellulosic degradation. At present, the CAZyme database is an important reference for annotating CAZymes in most microbial genomes or metagenomes (Cantarel et al., 2009; Zhu et al., 2016; Busk et al., 2017; Helbert et al., 2019).

All predicted coding genes were aligned with these nine databases with E-value cut-offs ≤1 × 10^−5^, identity ≥40%, and coverage >30%.

The gene clusters of secondary metabolites were predicted using antiSMASH version 7.0.1 with default parameters (Blin et al., 2023).

### 2.4 Phylogenomics analysis

To explore the evolutionary dynamics of *D. sinensis*, the genome sequences of 16 additional fungal species were downloaded from the National Center for Biotechnology Information (NCBI) (https://www.ncbi.nlm.nih.gov/genbank/) for phylogenomic analysis.

Single-copy orthologous genes from the 17 fungal species were inferred using OrthoFinder version 2.5.4 (Emms and Kelly, 2019) with the mafft option for subsequent multiple sequence alignment. On the basis of the resulting alignment, a maximum-likelihood tree was reconstructed using RAxML version 8.2.12 (Stamatakis et al., 2008). In addition, Timetree was used to calculate the divergence time among the 17 fungal species (Kumar et al., 2022).

Expansion and contraction of gene families were determined using CAFE version 4.2.1 (Han et al., 2013) with the following parameters: a cut-off *p*-value of 0.05; number of random samples = 1,000; the lambda value to calculate birth and death rates.

### 2.5 Phylogenetic analysis

The internal transcribed spacer (ITS) sequences in *Daedaleopsis* species were aligned using ClustalX 1.83 (Chenna et al., 2003) and optimized manually using BioEdit 7.0.5.3 (Hall, 1999) prior to phylogenetic analysis. Thereafter, maximum parsimony (MP) bootstrap analysis was performed using PAUP^*^ version 4.0 beta 10 to reveal the phylogenetics of *Daedaleopsis* species (Swofford, 2002). ITS sequence of *D. sinensis* was extracted as 559 bp from its genome data and then compared with other ten species in the same genus. At the same time, *Earliella scabrosa* was selected as an outgroup to construct the evolutionary tree (Li et al., 2016).

### 2.6 Comparative genomics analysis

To explore the dynamics of speciation in *D. sinensis*, the genome sequences of *D. sinensis* and *D. nitida* were aligned in pairs using MCScanX (Wang et al., 2012) and NGenomeSyn (He et al., 2023).

In addition, the numbers of genes encoding various families of CAZymes in *D. sinensis* and *D. nitida* were clustered in a heatmap using TBtools version 1.127 (Chen et al., 2020) with the log-scale option.

## 3 Results

### 3.1 Species identity

**FIGURE 1:**
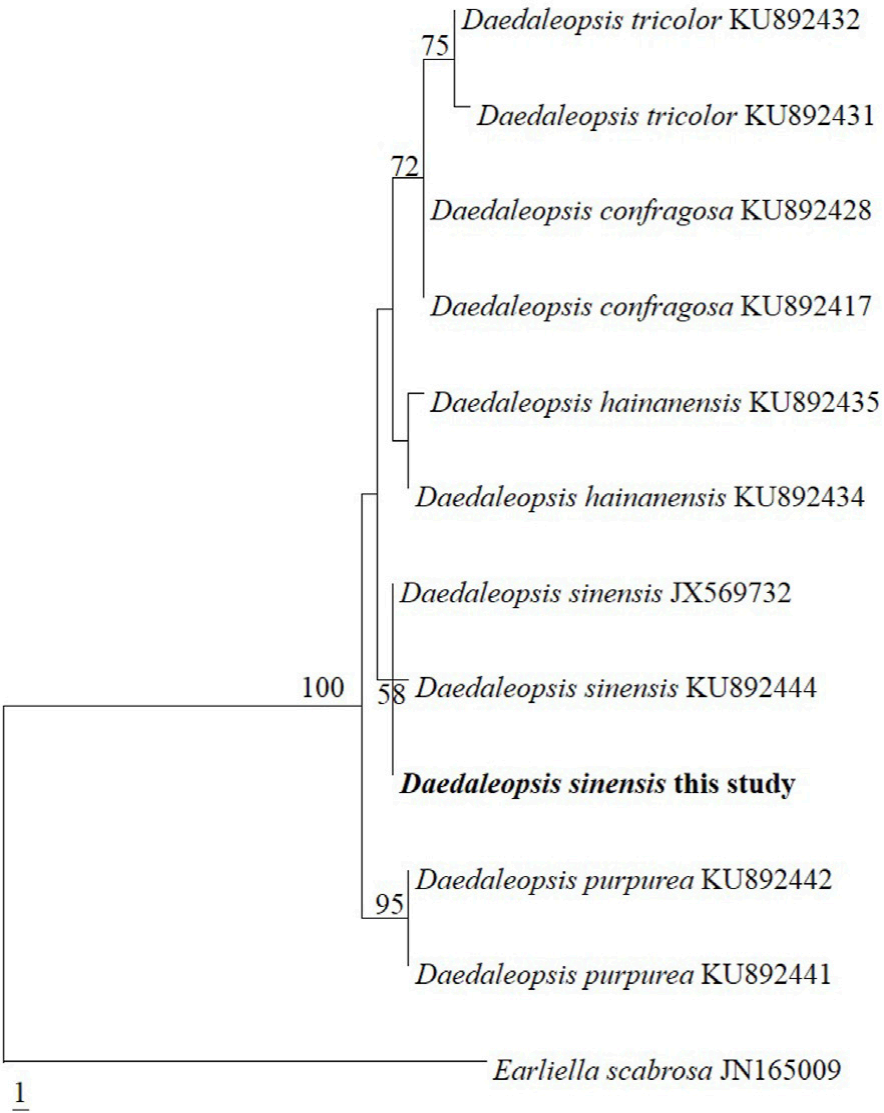
Phylogeny of *Daedaleopsis* constructed by maximum parsimony analysis based on internal transcribed spacer sequences.

Strain was identified as *D. sinensis* based on internal transcribed spacer-barcoding sequences. Figure 1 shows that the species used in the present study clustered with *D. sinensis* JX569732 and KU892444. Only one base difference from *D. sinensis* JX569732 and two base differences from *D. sinensis* KU892444, further confirming the strain used is *D. sinensis* based on phylogenetics.

### 3.2 Genome assembly and annotation

**FIGURE 2:**
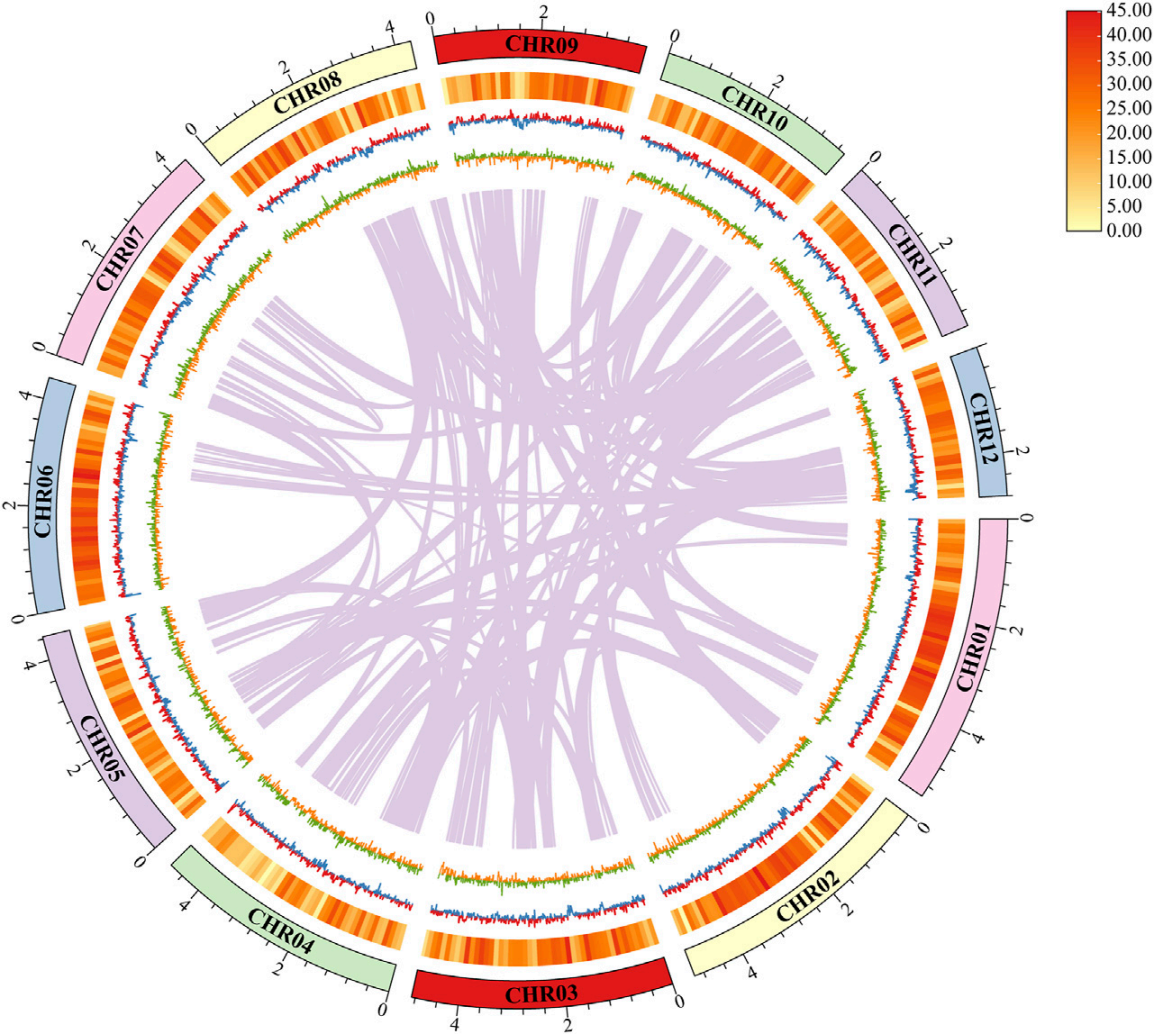
Characteristics of the *de novo* assembly genomic features of *Daedaleopsis sinensis*. From outside to inside: (1) Contigs (>2 Mb in length); (2) Gene density, a heat map was used to show the relative number of bases contained in genes; (3) GC content, calculated as the percentage of G + C in 1 kb non-overlapping windows. The inward blue part represents the GC content that is lower than the average genome GC content, whereas the outward red part represents the opposite; (4) GC skew, calculated as the percentage of (G−C)/(G + C) in 1 kb non-overlapping windows. The inward orange part represents (G−C)/(G + C) < 0, and the outward green part represents (G−C)/(G + C) > 0; (5) Genome duplication, the regions where sequence similarity exists are connected by purple lines.

The 51.67 Mb genome sequence was assembled from 6,113 Mb raw data (106 × genome coverage) and to 24 contigs with a GC content of 56.46% (Table 1). Of the 24 contigs, the longest one was 5.41 Mb, whereas the N50 length was 4.40 Mb (Table 2). The GC skew did not present an obvious distribution pattern in the whole genome (Figure 2). A K-mer analysis with a depth of 34 showed a 1.60% heterozygous rate of the genome. Collectively, the above information indicates the high quality of the genome sequence assembly of *D. sinensis*.

**TABLE 1:** *De novo* genome assembly and features of *Daedaleopsis sinensis*.

Contig	Characteristic	Genome	Characteristic
Total number	24	Genome assembly (Mb)	51.67
Total length (Mb)	51.67	Number of protein-coding genes	12,153
N50 length (Mb)	4.40	Average length of protein-coding genes (bp)	2,448.26
Max length (Mb)	5.41	Repeat size (Mb)	7.62
Coverage (%)	99.95	Transposable elements (Mb)	6.26
GC content (%)	56.46	tRNA (bp)	15,938

**TABLE 2:** Comparative genomics analyses of *Daedaleopsis sinensis* and *D. nitida*.

Characteristic		*D. sinensis*	*D. nitida*
Genome structure	Genome size (Mb)	51.67	41.8
	Number of contigs	24	33
	N50 length of contig (Mb)	4.4	3.2
	Protein-coding genes	12,153	14,978
	GC content (%)	56.46	56.2
Genes encoding CAZymes	AA	113	118
	CBM	7	7
	CE	33	35
	GH	237	249
	GT	73	67
	PL	18	18
	Total	481	494
Gene clusters of secondary metabolites	T1PKS	3	4
	NRPS	2	2
	NRPS-like	11	11
	terpene	24	21
	fungal-RiPP-like	5	5
	*β*-Lactone	1	1
	Total	46	44
Genes encoding cytochromes P450 (Top 10)	CYP51	128	262
	CYP620	99	166
	CYP53	58	112
	CYP4	48	72
	CYP504	22	41
	CYP505	20	39
	CYP102	17	28
	CYP78	16	28
	CYP125	15	33
	CYP512	15	30

A total of 12,153 protein-coding genes were predicted with an average gene length of 2,420.31 bp and an average coding sequences (CDS) length of 1,476.57 bp, whereas 280 non-coding RNAs (ncRNAs) were predicted, accounting for 23.93% of the whole genome sequence. The total length of repetitive sequences was 7.62 Mb (Table 2) with the tandem repeat sequences and the interspersed repeated sequences accounting for 7.73% and 12.11%, respectively, of the whole genome sequence. The length of the transposable elements was 4.60 Mb with long terminal repeats (LTR) and non-LTR accounting for 7.71% and 1.19%, respectively, of the whole genome sequence. Assessment of genome integrity using BUSCO showed 97.36% of complete genome, indicating that the vast majority of conserved genes were predicted to be relatively complete and implying that there was high confidence in the genome assembly and prediction.

### 3.3 KEGG pathways

The annotation of genes using the KEGG pathway database can expand understanding of the biological functions and interactions of genes. A total of 3,831 annotated genes had matched in the KEGG database and were assigned into five levels. Of the five main levels, metabolism was the largest (2,400 genes, 48.40%), followed by genetic information processing (826 genes, 16.66%), organismal systems (737 genes, 14.86%), cellular processes (665 genes, 13.41%), and environmental information processing (331 genes, 6.67%). Thus, there were genes corresponding to significant metabolic processes. Many pathways related to lignocellulosic degradation and some key enzymes secreted by *D. sinensis* were identified in the KEGG database.

Regarding the metabolism and biosynthesis of sugar-containing compounds, 22 enzymes encoded by 43 genes and involved in the biosynthesis of polysaccharides (starch and sucrose metabolism) were identified from *D. sinensis* (Supplementary Figure S1; Supplementary Table S1). Most of these enzymes were encoded by single- or double-copy genes, whereas *β*-glucosidase and cellulose 1,4-*β*-cellobiosidase were encoded by eight- and four-copy genes, respectively. In the glycolysis/gluconeogenesis pathway (Supplementary Figure S2; Supplementary Table S2), there were 24 enzymes encoded by 35 genes, some of which had double or multiple copies. Seventeen key enzymes involved in terpenoid backbone biosynthesis via the mevalonate pathway were identified from *D. sinensis* (Supplementary Figure S3). Of these 17 key enzymes, protein-*S*-isoprenylcysteine *O*-methyltransferase was encoded by two copies of the genes, whereas the remaining enzymes were each encoded by a single gene (Supplementary Table S3). Many genes involved in phenylalanine metabolism, benzoate degradation, toluene degradation, pyruvate metabolism, galactose metabolism, fatty acid degradation, and aminobenzoate degradation were identified in *D. sinensis*. For example, 24 key enzymes involved in the pyruvate metabolism pathway were identified (Supplementary Figure S4; Supplementary Table S4), with 37 genes encoding these enzymes, and 11 key enzymes involved in the phenylalanine metabolism pathway were identified, with 24 genes encoding these enzymes (Supplementary Figure S5; Supplementary Table S5).

### 3.4 Carbohydrate-active enzymes

**FIGURE 3:**
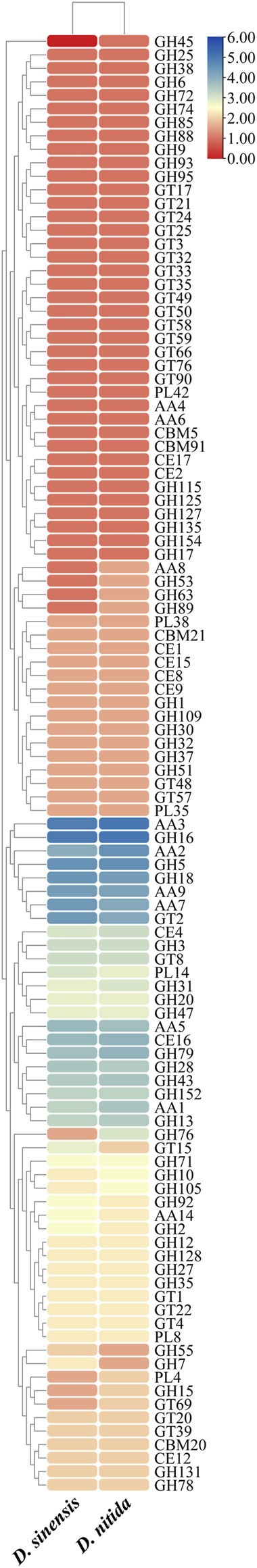
Heatmap of CAZymes classification statistics in *Daedaleopsis sinensis* and *D. nitida*. The *x* and *y*-axes represent species and CAZyme families, respectively. Rectangular blocks are colored with the logarithmic value of the number of genes encoding the CAZyme families; the color from dark red to dark blue indicates the increase in gene numbers.

The total number of genes encoding CAZymes among the two species of *Daedaleopsis* was more or less similar, as was the number of genes encoding each of the six classes of CAZymes (Table 2). Only a few differences could be concluded at the level of gene families encoding CAZymes (Figure 3). According to the number of genes belonging to different families of CAZymes, *D. sinensis* has a similar strategy to *D. nitida* for the utilization of woody substrates. From *D. sinensis*, 481 genes were assigned to the six classes of CAZymes, including 7 genes encoding carbohydrate-binding modules (CBMs), 33 carbohydrate esterases (CEs), 237 glycoside hydrolases (GHs), 73 glycosyltransferases (GTs), 18 polysaccharide lyases (PLs), and 113 auxiliary activities (AAs) (Table 2). Among the six classes, GHs were the dominant group (encoded by the most genes) and were mainly involved in the degradation of celluloses (GH1, GH3, GH5, GH6, GH7, GH9, GH12, GH51, and GH74), hemicelluloses (GH3, GH10, GH30, GH43, and GH51), pectins (GH28), chitins (GH18), and starches (GH31). The families each encoded by ten or more genes included AA2 (15), AA3 (29), AA5 (13), AA7 (20), AA9 (19), CE16 (13), GH16 (30), GH18 (21), GH28 (11), GH43 (10), GH5 (22), GH79 (12), and GT2 (20). The types and quantities of enzymes found in *D. nitida* were similar to those found in *D. sinensis* (Table 2; Supplementary Table S6).

### 3.5 Cytochrome P450 monooxygenases

**FIGURE 4:**
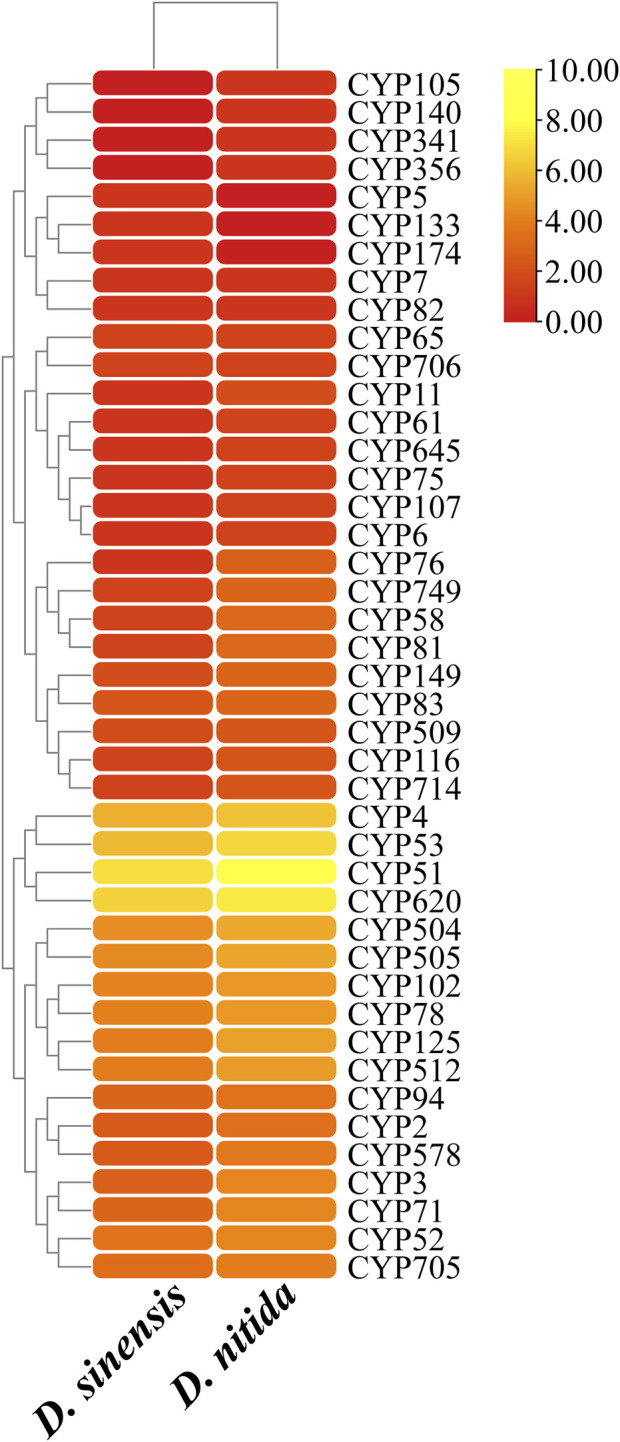
CYPs statistics in *Daedaleopsis sinensis* and *D. nitida*. The *x* and *y*-axes represent species and CYP families, respectively. The rounded rectangular blocks are colored with the logarithmic values of the number of genes encoding the CYPs; the color from red to yellow indicates the increases in gene numbers.

CYPs play a variety of roles in fungi, including detoxification, heterobiomass degradation, and biosynthesis of secondary metabolites. Genes encoding CYPs comprise 4.3% of the CDS of the genome. After comparison with the CYP database, 524 genes encoding 39 CYPs were identified in *D. sinensis* (Table 2; Figure 4). In *D. nitida*, 40 CYPs encoded by 992 genes were identified. The most abundant CYP families in *D. sinensis* were CYP51, CYP620, CYP53, and CYP4.

### 3.6 Gene cluster

A total of 44 gene clusters were predicted from *D. sinensis*. Of them, 23 encode terpene synthases (TS), 3 encode iterative type I polyketide synthases (T1PKS), 2 encode nonribosomal peptide synthetases (NRPS), 11 encode NRPS-like fragment, and 5 encode fungal unspecified ribosomally synthesized and post-translationally modified peptide product (fungal-RiPP-like) (Table 2). In its congeneric species *D. nitida*, 44 secondary metabolite clusters were predicted, of which 21 were TS, 4 were T1PKS, and the rest were the same as *D. sinensis*.

### 3.7 Phylogenomics

**FIGURE 5:**
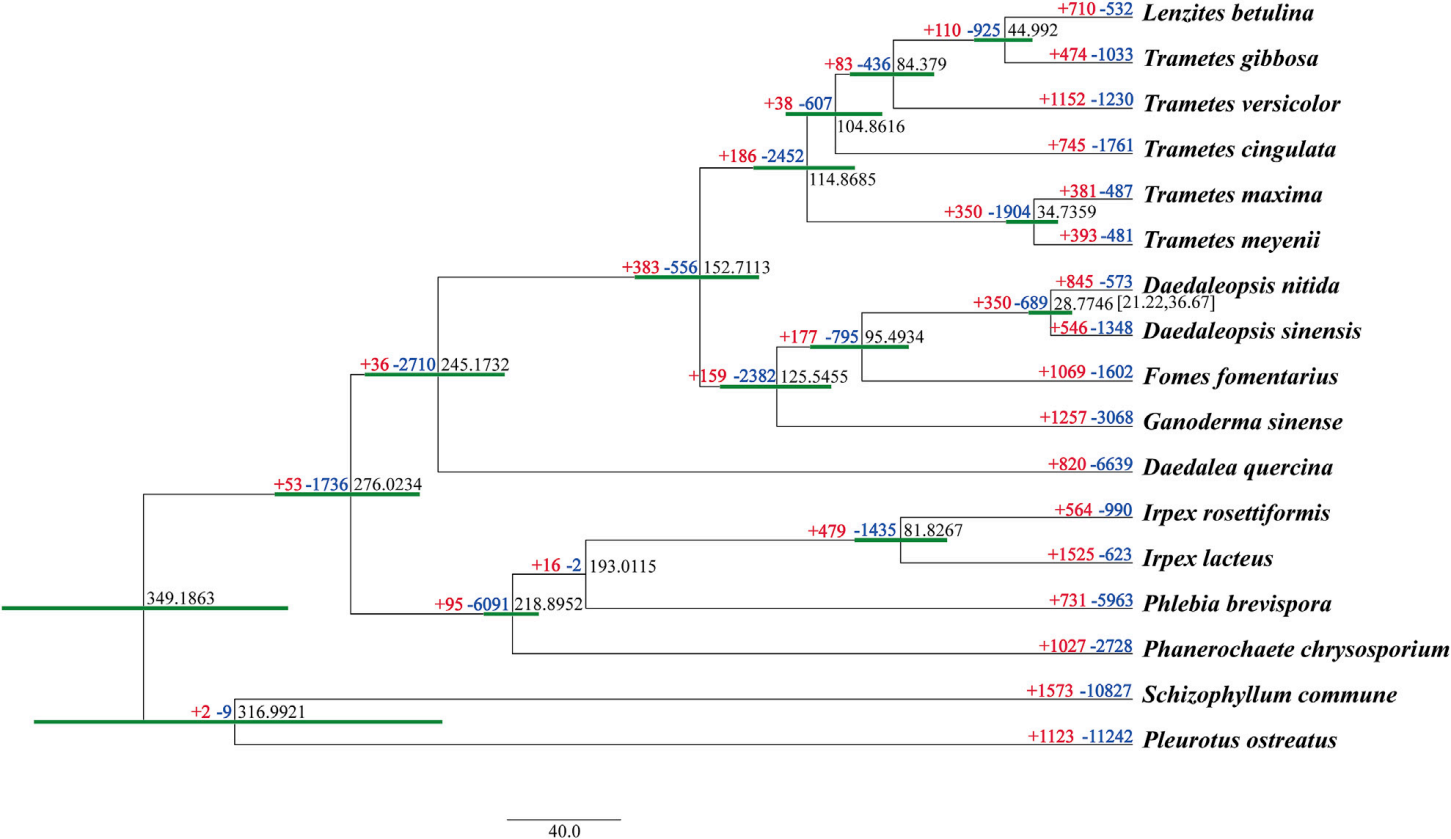
Maximum clade credibility tree inferred from 1,402 single-copy orthologous genes. All nodes received full bootstrap support. The divergence time is labeled as the mean crown age for each node, and the 95% highest posterior density is also given within the *Daedaleopsis* clade. The numbers of gene family expansions and contractions in each species are labeled after plus (red) and minus (blue) symbols, respectively.

A total of 2,260 orthologous groups, including 1,402 single-copy genes, were identified from all 17 studied fungal species. The phylogenomic tree inferred from an alignment of the 1,402 single-copy orthologous genes with 530,910 characters of amino acid residues delimited the phylogenetic relationships among the 17 species with full bootstrap support (Figure 5).

Besides two species in *Daedaleopsis*, other white rot fungal species, *viz*. *Trametes versicolor*, *Fomes fomentarius*, *Ganoderma sinense*, *Irpex rosettiformis*, *Pleurotus ostreatus*, and *P. chrysosporium*, were also phylogenetically separated. Of the species in *Daedaleopsis*, *D. sinensis* and *D. nitida*, which occurred in a mean crown age of 28.77 Mya with a 95% highest posterior density of 21.22–36.67 Mya, had a close phylogenetic relationship. This is also the first time that the pairwise divergence time between these two species has been given. In the evolutionary process of the 17 sampled fungal species, gene family contraction occurred more commonly than gene family expansion (Figure 5). Regarding *Daedaleopsis*, 546 and 845 gene families had expanded in *D. sinensi*s and *D. nitida*, respectively, whereas 1,348 and 573gene families had contracted in *D. sinensi*s and *D. nitida*, respectively. Of these gene families, 46 (10 expanded and 36 contracted), and 59 (52 expanded and 7 contracted) in *D. sinensis* and *D. nitida*, respectively, experienced a rapid evolution.

### 3.8 Comparative genomics

**FIGURE 6:**
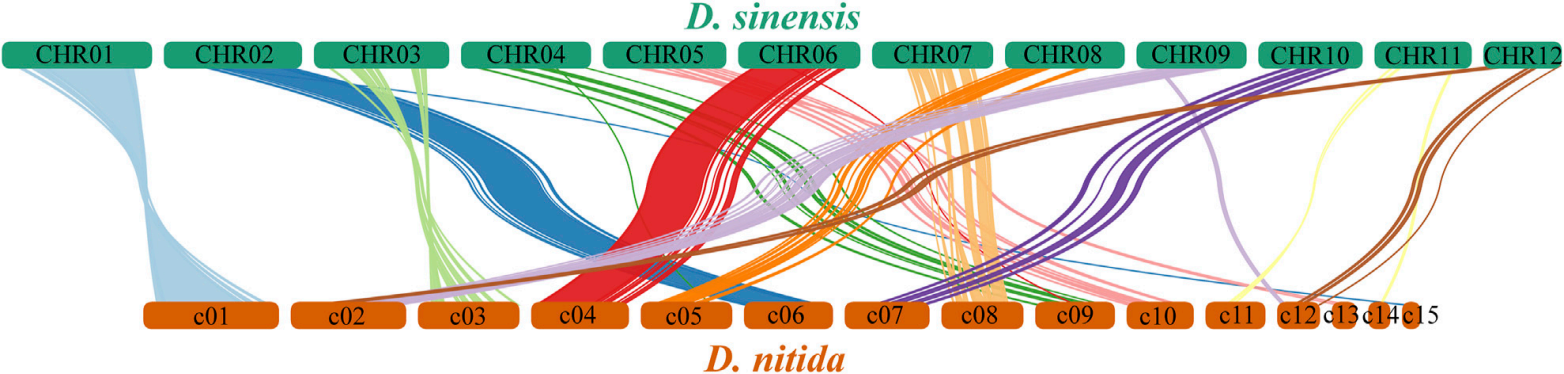
Distribution and arrangement of homologous genes between *Daedaleopsis sinensis* and *D. nitida*.

Arrangement of the homologous genes or sequences between contigs >2.5 Mb in *D. sinensis* and contigs >0.6 Mb in *D. nitida* (Figure 6). Figure 6 shows only the connecting lines with similarity >5,000. Comparisons of homology allow for the study of evolutionary relationships between the two species.

**FIGURE 7:**
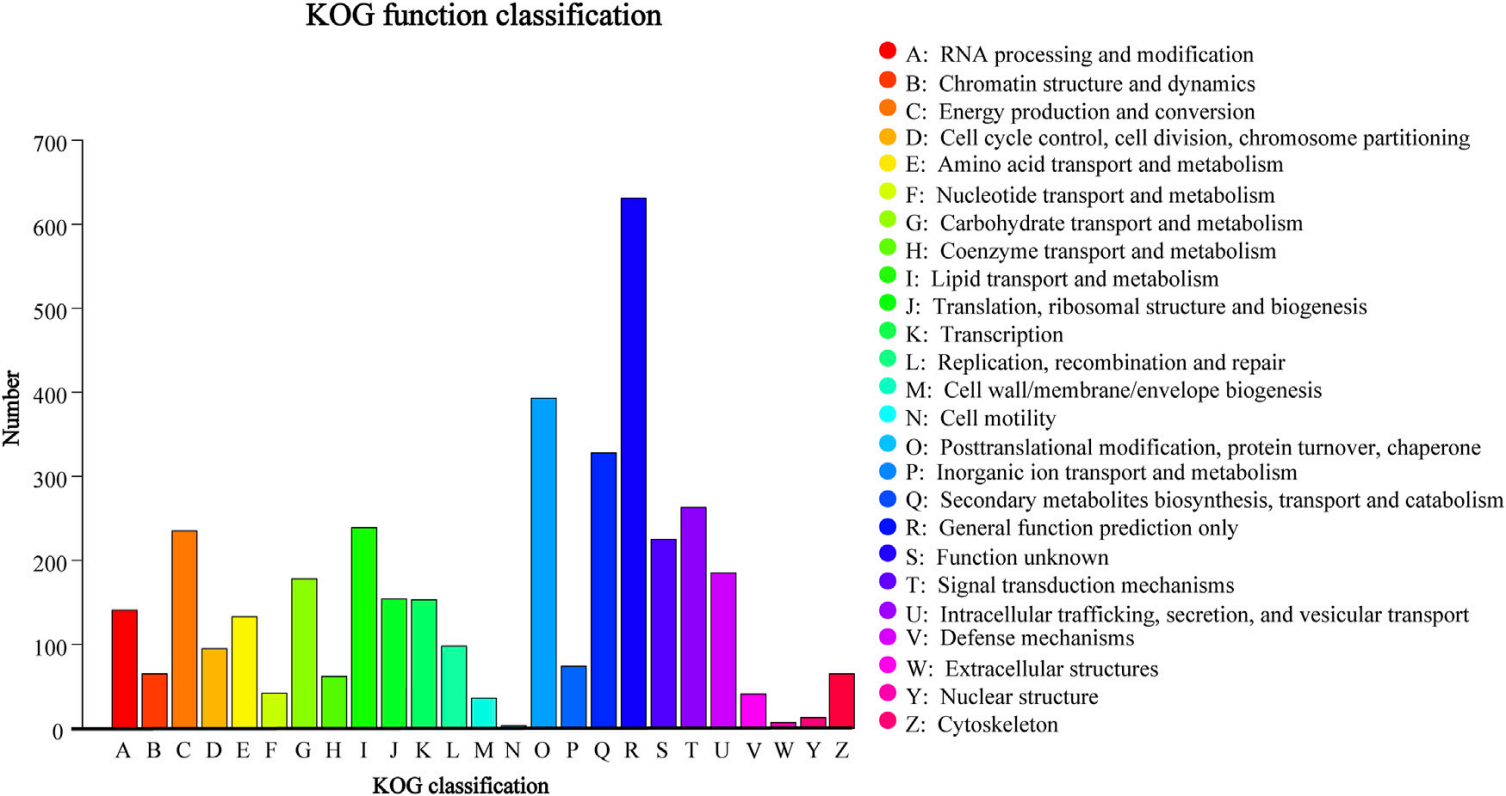
KOG classification in *Daedaleopsis sinensis*.

Using the KOG database, 3,367 (27.71%) genes in *D. sinensis* were assigned to KOG categories (Figure 7). The most gene-rich KOG classifications were “general function prediction only,” “posttranslational modification, protein turnover, chaperones,” “secondary metabolites biosynthesis, transport, and catabolism,” and “signal transduction mechanisms.”

**FIGURE 8:**
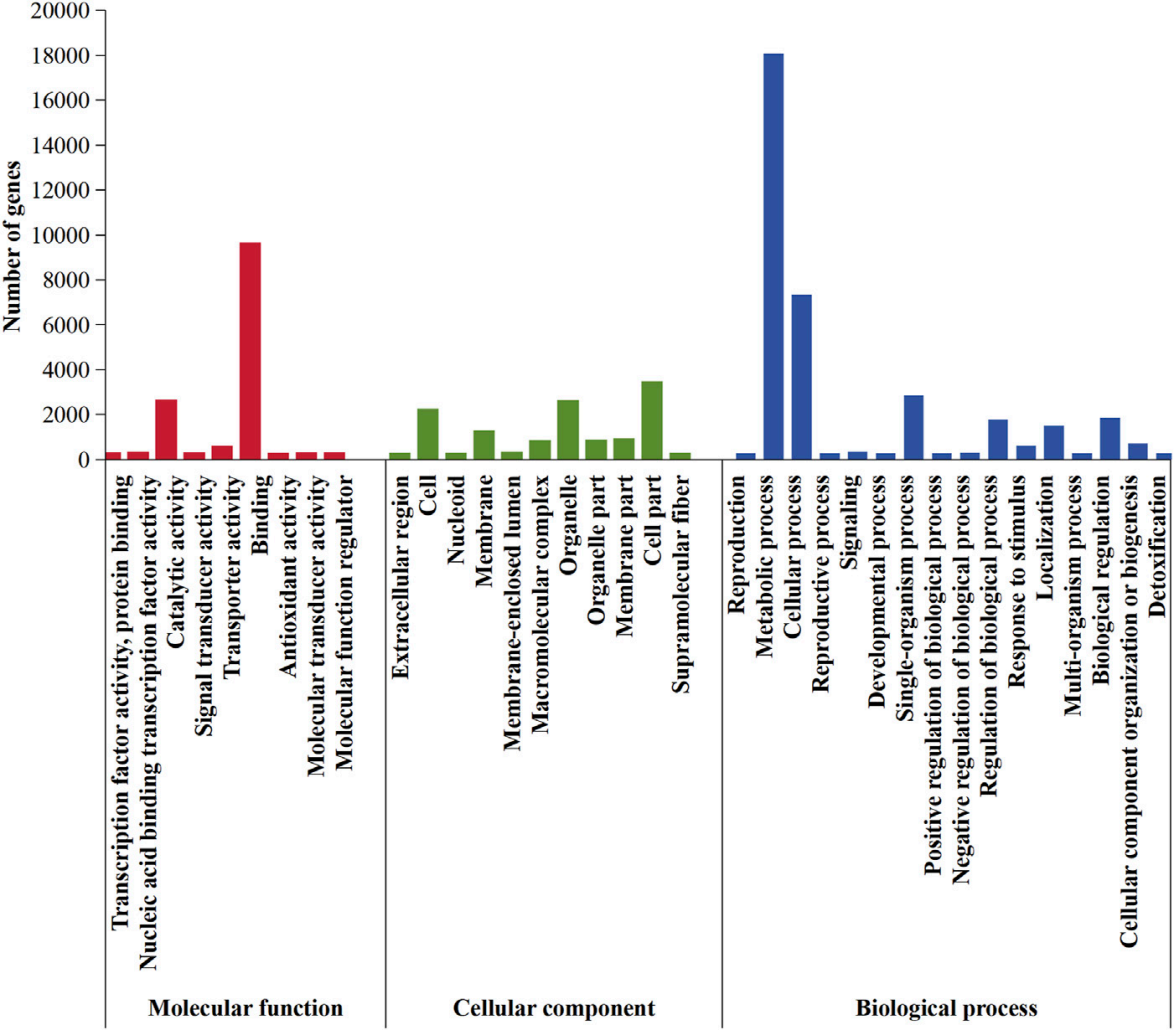
GO classification in *Daedaleopsis sinensis*.

According to the GO database, 5,612 genes that accounted for 46.18% of the entire genome were distributed in the three functional categories of biological process, cellular components, and molecular function (Figure 8). Among biological processes, 17,835 genes were involved in “metabolic activities,” followed by “cellular process” (7,116 genes), “single-organism process” (2,627 genes), and “biological regulation” (1,617 genes) functions. Of the genes with products predicted to be involved in molecular function, 9,380, 2,407, and 329 were involved in binding, catalytic activity, and transporter activity, respectively. For cellular components, 3,224, 2,389, 1,996, and 1,029 genes were involved in the formation process of cell part, organelle, cell, and membrane, respectively.

In the two species of *Daedaleopsis*, the number of gene clusters involved in the synthesis of NRPS, NRPS-like, fungal-RiPP-like, and *β*-lactone was similar, and *D. sinensis* had the highest and the lowest numbers of gene clusters, which were involved in the syntheses of terpene and *β*-lactone, respectively (Table 2). In *D. sinensis*, there were 24 gene clusters encoding terpenes, which are key biosynthetic enzymes; 3 gene clusters encoding T1PKS, which are associated with the biosynthesis of polyketides; and 2 gene clusters encoding NRPSs, which are modular enzymes.

## 4 Discussion

### 4.1 Whole genome sequencing of *D. sinensis*

The genome sequencing and annotation of *D. sinensis* are crucial for its function and comparative genomics research. Following accurate species identification, this study presented the first whole genome sequencing of *D. sinensis*. Information on *de novo* genome assembly and characterization of *D. sinensis* is summarized in Table 1.

### 4.2 Cytochrome P450 monooxygenases

The most abundant CYP families in *D. sinensis* are CYP51, CYP620, CYP53, CYP4, CYP504, CYP505, CYP102, and CYP125. CYP51 is a structurally and functionally conserved fungal P450 family (Yu et al., 2020), and is widely distributed in different biological kingdoms, being found in animals, plants, fungi, yeast, lower eukaryotes, and bacteria (Yoshida et al., 2000; Lepesheva and Waterman, 2004). CYP51 is a key enzyme in biosterol synthesis (Yoshida et al., 2000) and can catalyze the 14*α*-methyl hydroxylation of the precursor of sterol (Waterman and Lepesheva, 2005; Lepesheva and Waterman, 2007). CYP51 has become an important target for cholesterol-lowering drugs, antifungal drugs, and herbicides (Lepesheva and Waterman, 2004). Genes in the CYP53 family are involved in degradation or detoxification of benzoate and its derivatives (Crešnar and Petrič, 2011; Yu et al., 2020). CYP505 genes are involved in *ω*-1 to *ω*-3 carbon hydroxylation of fatty acids (Nakayama et al., 1996; Bao et al., 2013). Search on biosynthesis of secondary metabolites showed that the CYP620 family is related to “carbohydrate metabolism-amino sugar and nucleotide sugar metabolism” and may play a role in terpenoid synthases (Yu et al., 2020). CYP504 family members are linked to the degradation of phenylacetate and its derivatives (Ferrer-Sevillano and Fernández-Cañón, 2007; Crešnar and Petrič, 2011).

### 4.3 Carbohydrate-active enzymes

According to the CAZyme database, GH genes involved in the degradation of glucoside bonds between sugars and sugars or between sugars and non-sugar groups were the most abundant CAZyme groups in *D. sinensis*, accounting for 49.27% of the total CAZyme gene sequences (Table 2). The second largest group with a proportion of 23.49% was the coenzyme family enzymes (AAs), which are involved in other CAZymes (some families are also involved in the degradation of lignin). Genes encoding GTs, which catalyze the biosynthesis of monosaccharides, disaccharides, oligosaccharides, and polysaccharides, accounted for 15.18% of the total CAZyme gene sequences in *D. sinensis*. CEs, which are responsible for sugar modification, and CBMs, which promote anchoring of lignocellulose-degrading enzymes to the corresponding substrate, accounted for 6.86% and 1.46%, respectively. The proportion of PLs, which are involved in the cleavage of polysaccharide aldehyde polyglycan chains, was 3.74%. In summary, the cumulative proportion of GH and PL genes responsible for the degradation of carbohydrates in *D. sinensis* was as high as 53.01%, and this reflects, to some extent, the ability of *D. sinensis* to degrade lignocellulose.

#### 4.3.1 Enzymes involved in cellulose degradation

Cellulose is a linear polymer composed of 500–15,000 glucose molecules linked via *β*-1,4-glucoside bonds. Due to it comprising the largest proportion of lignocellulosic biomass (approximately 45% of dry weight), cellulose is considered the most valuable part of lignocellulosic biomass. The complete hydrolysis of cellulose is accomplished by the synergistic action of three different types of cellulases: endo-1,4-*β*-glucanases (EC 3.2.1.4), exo-1,4-*β*-glucanases (EC 3.3.1.91), and *β*-glucosidases (EC 3.2.1.21) (Zhou et al., 2017). Cellulose-degrading enzymes typically contain one or more CBMs (Arfi et al., 2014). In the CAZyme database, the most of these enzymes belong to three families: GH3, GH5, and GH9. Endoglucanase hydrolyzes the internal glycosidic bonds in the cellulose chain, releasing glucose, cellobiose, oligosaccharides, and other products, while exobiose hydrolase attacks the highly ordered crystalline and amorphous regions of the fiber, and subsequently releases cellobiose. The cellobiose and oligosaccharides released by these two enzymes are further hydrolyzed to glucose by *β*-glycosidases from the GH1, GH3, GH5, GH9, and GH116 families. Enzymes from the GH3 family can remove glycosylated residues from the non-reducing end of carbohydrates and the side chain of hemicellulose and convert them into small substrates, releasing oligosaccharides and monosaccharides that can provide energy for the growth of host microorganisms (Harvey et al., 2000; Lacerda Júnior et al., 2017). The number of the genes of GH5 and GH12 families in *D. sinensis* was higher in the GHs families (Figure 3), therefore the activity of endoglucanase may be stronger, along with its ability to degrade the amorphous region of cellulose. GH5 family is the most conserved cellulase family, also known as “cellulase family A” (Henrissat et al., 1989), and can hydrolyze glycosidic bonds through an acid/base retention mechanism. Its tertiary structure presents a *a* (*β*/*α*)_8_ barrel-folded conformation. The members in GH5 family have a wide range of substrate specificity, conferring their extensive industrial potential including alteration of the taste of fruits and vegetables (Marlatt et al., 1992), improvement of the aroma of wine (Caldini et al., 1994), and enhancement the ability of animals to digest and absorb feeds (Bedford and Classen, 1992). GH12 has the smallest molecular weight in the GH family, with catalytic domain but without CBM region. Amino acid triplet structures are found in most enzymes of the GH12 family, which make up for the absence of CBM region to some extent (Prates et al., 2013). Many glycoside hydrolases in GH12 family have been used in transglycosylation for enzymatic synthesis of macromolecular materials as well as bio-polishment and decolorization to improve the softness and surface appearance of cotton fabrics (Gusakov et al., 2001; Kobayashi et al., 2010; Tanaka et al., 2010). Obviously, the large numbers of GH5 and GH12 genes in *D. sinensis* also suggested potential industrial values of this species. The number of GH1 and GH3 genes was relatively higher among the genes encoding CAZymes in *D. sinensis* (Figure 3). Consequently, the activity of *β*-1,4-glucosidase in *D. sinensis* is relatively strong, and the decomposition ability of the cellulose crystallization region is fascinating. Similar to other wood-decaying fungi, the degradation of cellulose by *D. sinensis* mainly depends on the synergistic interaction between different enzyme systems. In general, *D. sinensis* has a strong ability to degrade cellulose.

#### 4.3.2 Enzymes involved in hemicellulose degradation

Hemicellulose has a complex structure, among which xylan and mannan are the most abundant constituents (Dhawan and Kaur, 2007). The enzymes involved in xylan and mannan hydrolysis mainly include endo-*β*-1,4-xylanases, *β*-1,4-xylosidases, and exo-*β*-1,4-glucanases. The complex structure of hemicellulose means that the synergistic participation of GHs and CEs is required for complete degradation of the plant polysaccharide. GH43, GH10, and GH78 families are the main families containing hemicellulases, among which GH43 family is the most abundant. The enzymes of this family usually have a variety of hemicellulolytic activities and can generally degrade xylan and araban, which could be applied in starch processing, bioenergy manufacturing, feed enzyme preparation, wine production, and pharmaceutical synthesis (Grootaert et al., 2007; Ferrer et al., 2012). In *D. sinensis*, the number of genes encoding GH43 family enzymes was also relatively high. *β*-xylosidase, which is a key enzyme in xylan degradation, has a crucial role in the conversion of hemicellulose substrates and important application potential in substrate recognition and lignocellulosic resource conversion (Lagaert et al., 2014; Rohman et al., 2019). The reported *β*-xylosidases from fungi are predominantly concentrated in the GH3 and GH43 families, with those from filamentous fungi mainly found in the GH3 family (Lagaert et al., 2014; Rohman et al., 2019). Notably, there were more genes encoding GH3 family enzymes in *D. sinens*is. In addition, CEs can degrade hemicellulose by catalyzing the hydrolysis of acetyl groups on xylans (Armendáriz-Ruiz et al., 2018). Two genes encoding glucuronyl esterase (CE15) were identified in *D. sinens*is. A complex cross-linking structure exists between lignin and hemicellulose through ester and ether bonds (Balakshin et al., 2011). Glucuronic acid (GlcA) is methylated to 4-*O*-methyl-_D_-glucuronic acid (MeGlcA). MeGlcA is linked to xylan via 1–2 on one side, whereas on the other side its carboxyl group forms an ester bond with the alcohol hydroxyl group of lignin, which in turn forms a skeleton structure in which the cell wall becomes hardened (Spániková and Biely, 2006). CE15 can hydrolyze the ester bond between MeGlcA and lignin (Monrad et al., 2018). Thus, CE15 is instrumental in the dissociation of lignin from cellulose and hemicellulose. Collectively, these related enzymes provide some evidence of the ability of *D. sinensis* to degrade hemicellulose.

#### 4.3.3 Enzymes involved in lignin degradation

Lignin is a highly heterogeneous and resistant aromatic polymer that accounts for 10%–30% of the total lignocellulosic biomass (Wang et al., 2015). White rot fungi catalyze the initial depolymerization of lignin by secreting an array of oxidases and peroxidases that generate highly reactive and nonspecific free radicals, which in turn undergo a complex series of spontaneous cleavage reactions (Kirk and Farrell, 1987; Martinez et al., 2004). The complete degradation of lignin requires two groups of enzymes: lignin-modifying enzymes (LMEs) and lignin-degrading auxiliary enzymes (LDAs) (Janusz et al., 2017). LMEs mainly belong to the AA1 family and AA2 family in the CAZyme database. LDAs themselves cannot degrade lignin, but they can use O_2_ to produce H_2_O_2_, accompanied by the oxidation of aromatic alcohols and glyoxals or reduction of carbohydrates. The H_2_O_2_ produced can support LMEs or trigger non-enzymatic Fenton reactions that degrade lignin (Sweeney and Xu, 2012). AA1 enzymes are multicopper oxidases that use diphenols and related substances as donors with oxygen as the acceptor. The AA1 family is currently divided into three subfamilies: laccases, ferroxidases, and laccase-like multicopper oxidases. The AA2 family contains class II lignin-modifying peroxidases. AA2 enzymes are secreted heme-containing enzymes that use H_2_O_2_ or organic peroxides as electron acceptors to catalyze numerous oxidative reactions. In addition, AA3 enzymes are flavoproteins containing a flavin-adenine dinucleotide-binding domain. The AA3 family is one of the main families that contain lignin-degrading enzymes and belongs to the glucose-methanol-choline oxidoreductases family, which could be widely used in waste-to energy regenerating and biosensor manufacturing (Henske et al., 2018; Sützl et al., 2018; Sugano et al., 2021). AA3 is a powerful family of redox enzymes, all of whose members have similar structural characteristics, and can assist other AA family enzymes in catalysis through its reaction products or participate in the role of GHs in lignocellulose degradation. Besides, AA4, AA5, AA6, AA7, and AA9 families all have a certain amount of lignin degradation related enzymes. The abundance of enzymes encoding AA2, AA3, and AA7 families in *D. sinensis* is high. The above situation indicates that *D. sinensis* contains a large number of genes encoding lignin-degrading enzymes, reflecting the lignin-degrading ability of *D. sinensis*.

#### 4.3.4 Enzymes involved in pectin degradation

The modification and disassembly of pectin are executed by various hydrolytic enzymes (Mahmood et al., 2021). In the genome of *D. sinensis*, the number of GH28 (polygalacturonase, PG) genes encoding pectin decomposing enzyme was as high as 11, and the number of CE8 (pectin methylesterase, PME) encoding gene was only 2. Among the hydrolases, PGs are major pectin hydrolytic enzymes that catalyze the hydrolysis of the *α*-(1→4) and _D_-GlcA linkage in homogalacturonan, leading to cell separation (Ke et al., 2018). PME can hydrolyze methyl ester group in pectin, release methanol, and reduce the degree of methylation of pectin. PME is an important pectin enzyme and has a wide application prospect in food processing and paper production (Wang et al., 2020). PME and PG together can significantly enhance the clarifying effect of fruit juice (Tu et al., 2013). Other pectin decomposing enzyme genes (CE12, GH53, GH78, GH88, GH105, and PL4) were present in *D. sinensis*. This indicates that *D. sinensis* tends to hydrolyze all components in pectin.

### 4.4 KEGG metabolic pathways related to lignocellulosic degradation

According to the KEGG database analysis of *D. sinensis* data, 3,831 genes are involved in nearly 120 metabolic pathways. These pathways mainly include carbon metabolism, fatty acid biosynthesis and degradation, amino and nucleotide sugar metabolism, terpenoid metabolism, starch and sucrose metabolism, metabolism of cofactors and vitamins, methane metabolism, polycyclic aromatic hydrocarbon degradation, pyruvate metabolism, phenylalanine metabolism, and carbohydrate metabolism. Among them, the metabolism of sugar-containing compounds includes the tricarboxylic acid cycle and the classic pentose phosphate pathway. In addition, fructose, mannose, and galactose metabolism pathways are predicted. Studies have shown that the degradation of lignin by white rot fungi generally belongs to secondary metabolism, whereas the degradation of cellulose and hemicellulose exists in both primary and secondary metabolic stages (Zhao et al., 2019). The KEGG results indicate that substrate and energy metabolism and trophic growth are among the major biological processes carried out by *D. sinensis*, which is consistent with the reality that catabolism and trophic growth are the predominant life activities of *D. sinensis* itself as a degrader. The genome data suggested that *D. sinensis* possesses several carbon metabolic pathways and glucose metabolic pathways related to the degradation of cellulose and hemicellulose. There are 35 genes in the genome involved in glycolysis or gluconeogenesis pathways, and 43 genes involved in starch and sucrose metabolism pathways. The starch and sucrose metabolic pathways are closely related to the degradation of cellulose. In the pathways, a total of 11 genes encoding cellulase were identified. Among them, eight genes encode *β*-glucosidase, two genes encode exoglucanase, and one gene encodes endoglucanase. These annotated cellulase genes are involved in the transformation of cellulose into _D_-glucose (Supplementary Figure S1). Cellulose can be converted to cellodextrin by the action of endoglucanase, and then to cellobiose with the participation of *β*-glucosidase (or a combination of endoglucanase and exglucanase), which is hydrolyzed to _D_-glucose by the action of *β*-glucosidase. The benzoic acid metabolism pathway is the main metabolic pathway in the process of lignin degradation, but this has been less studied in fungi and mainly reported in bacteria (Egland et al., 1997). In *D. sinensis*, the pathways associated with lignin degradation include those associated with the degradation of aromatic compounds, such as phenylalanine metabolism, benzoate degradation, toluene degradation, pyruvate metabolism, galactose metabolism, fatty acid degradation, and aminobenzoate degradation.

## 5 Conclusion

The whole genome sequence of *D. sinensis* was obtained for the first time. Genomic analysis shows that potential ability of *D. sinensis* to degrade lignocellulosic materials. The analysis of the lignocellulose degradation capacity of *D. sinensis* is vital for future wide-ranging applications of this fungus. Further studies on *D. sinensis* should focus on its metabolic pathways as well as enzymatic analysis. In addition, subsequent transcriptome and metabolome data will further facilitate the application of *D. sinensis*. A comprehensive understanding of the *D. sinensis* genome is expected to pave the way for applications of this fungus in industrial production.

## Data Availability

The original contributions presented in the study are publicly available. This data can be found here: https://www.ncbi.nlm.nih.gov/bioproject/PRJNA1030494/.
