# Supplementary material for: Whole genome sequencing and annotation of Daedaleopsis sinensis, a wood-decaying fungus significantly degrading lignocellulose
Source: Front Bioeng Biotechnol. 2024 Jan 16;11:1325088. doi: 10.3389/fbioe.2023.1325088 (PMC10826855; doi:10.3389/fbioe.2023.1325088)
Supplement: Supplementary file 1 [file DataSheet1.doc]

**TABLE S1 Putative genes involved in** **the biosynthesis of polysaccharides (starch and sucrose metabolism) in Daedaleopsis sinensis.**

| **Gene name and definition** | **Enzyme code** | **KO term** | **Gene ID** |
| --- | --- | --- | --- |
| PYG, glgP; glycogen phosphorylase | 2.4.1.1 | K00688 | A04.229 |
| GYS; glycogen synthase | 2.4.1.11 | K00693 | A11.272 |
| otsA; trehalose 6-phosphate synthase | 2.4.1.15 | K00697 | A08.287 |
| TPS; trehalose 6-phosphate synthase / phosphatase | 2.4.1.15, 3.1.3.12 | K16055 | A12.166 |
| GBE1, glgB; 1,4-*α*-glucan branching enzyme | 2.4.1.18 | K00700 | A06.455 |
| GYG1, GYG2; glycogenin | 2.4.1.186 | K00750 | A01.857 |
| GYG1, GYG2; glycogenin | 2.4.1.186 | K00750 | A03.342 |
| GYG1, GYG2; glycogenin | 2.4.1.186 | K00750 | A05.694 |
| AGL; glycogen debranching enzyme | 2.4.1.25,3.2.1.33 | K01196 | A05.806 |
| E.2.4.1.34; 1,3-*β*-glucan synthase | 2.4.1.34 | K00706 | A02.700 |
| E.2.4.1.34; 1,3-*β*-glucan synthase | 2.4.1.34 | K00706 | A03.962 |
| HK; hexokinase | 2.7.1.1 | K00844 | A03.1024 |
| HK; hexokinase | 2.7.1.1 | K00844 | A06.712 |
| UGP2, galU, galF; UTP--glucose-1-phosphate uridylyltransferase | 2.7.7.9 | K00963 | A07.440 |
| AMY, amyA, malS; *α*-amylase | 3.2.1.1 | K01176 | A02.460 |
| AMY, amyA, malS; *α*-amylase | 3.2.1.1 | K01176 | A04.43 |
| AMY, amyA, malS; *α*-amylase | 3.2.1.1 | K01176 | A08.146 |
| malZ; *α*-glucosidase | 3.2.1.20 | K01187 | A01.122 |
| malZ; *α*-glucosidase | 3.2.1.20 | K01187 | A01.1400 |
| malZ; *α*-glucosidase | 3.2.1.20 | K01187 | A02.103 |
| malZ; *α*-glucosidase | 3.2.1.20 | K01187 | A02.74 |
| bglX; *β*-glucosidase | 3.2.1.21 | K05349 | A01.548 |
| bglX; *β*-glucosidase | 3.2.1.21 | K05349 | A01.591 |
| bglX; *β*-glucosidase | 3.2.1.21 | K05349 | A02.347 |
| bglX; *β*-glucosidase | 3.2.1.21 | K05349 | A02.59 |
| bglX; *β*-glucosidase | 3.2.1.21 | K05349 | A04.130 |
| bglX; *β*-glucosidase | 3.2.1.21 | K05349 | A06.1236 |
| E.3.2.1.21; *β*-glucosidase | 3.2.1.21 | K01188 | A03.883 |
| E.3.2.1.21; *β*-glucosidase | 3.2.1.21 | K01188 | A10.632 |
| INV, sacA; *β*-fructofuranosidase | 3.2.1.26 | K01193 | A05.912 |
| INV, sacA; *β*-fructofuranosidase | 3.2.1.26 | K01193 | A05.919 |
| TREH, treA, treF; *α*,*α*-trehalase | 3.2.1.28 | K01194 | A08.465 |
| TREH, treA, treF; *α*,*α*-trehalase | 3.2.1.28 | K01194 | A09.167 |
| SGA1; glucoamylase | 3.2.1.3 | K01178 | A09.759 |
| E.3.2.1.4; endoglucanase | 3.2.1.4 | K01179 | A08.576 |
| E.3.2.1.4; endoglucanase | 3.2.1.4 | K01179 | A10.641 |
| INV, sacA; *β*-fructofuranosidase | 3.2.1.58 | K01210 | A05.912 |
| INV, sacA; *β*-fructofuranosidase | 3.2.1.58 | K01210 | A05.919 |
| CBH1; cellulose 1,4-*β*-cellobiosidase | 3.2.1.91 | K01225 | A01.1346 |
| CBH1; cellulose 1,4-*β*-cellobiosidase | 3.2.1.91 | K01225 | A01.853 |
| CBH1; cellulose 1,4-*β*-cellobiosidase | 3.2.1.91 | K01225 | A05.772 |
| CBH2, cbhA; cellulose 1,4-*β*-cellobiosidase | 3.2.1.91 | K19668 | A02.1062 |
| GPI, pgi; glucose-6-phosphate isomerase | 5.3.1.9 | K01810 | A11.269 |
| pgm; phosphoglucomutase | 5.4.2.2 | K01835 | A02.457 |
| TSL1, TPS3; trehalose 6-phosphate synthase complex regulatory subunit | 3.1.3.12 | K22337 | A04.303 |

**Table S2 Putative genes involved in the pathway of glycolysis / gluconeogenesis in *Daedaleopsis sinensis*.**

| **Gene name and definition** | **Enzyme code** | **KO term** | **Gene ID** |
| --- | --- | --- | --- |
| ACSS, acs; acetyl-CoA synthetase | 6.2.1.1 | K01895 | A03.858 |
| adhP; alcohol dehydrogenase, propanol-preferring | 1.1.1.1 | K13953 | A02.230 |
| adhP; alcohol dehydrogenase, propanol-preferring | 1.1.1.1 | K13953 | A08.294 |
| adhP; alcohol dehydrogenase, propanol-preferring | 1.1.1.1 | K13953 | A12.537 |
| AKR1A1, adh; alcohol dehydrogenase [NAD(P)^+^] | 1.1.1.2 | K00002 | A07.128 |
| NAD(P)^+^ | 1.2.1.5 | K00129 | A10.768 |
| ALDH; aldehyde dehydrogenase (NAD^+^) | 1.2.1.3 | K00128 | A01.47 |
| ALDH; NAD^+^ | 1.2.1.3 | K00128 | A06.747 |
| ALDH7A1; aldehyde dehydrogenase family 7 member A1 | 1.2.1.3 | K14085 | A03.752 |
| DLAT, aceF, pdhC; pyruvate dehydrogenase E2 component (dihydrolipoamide acetyltransferase) | 2.3.1.12 | K00627 | A01.1006 |
| DLD, lpd, pdhD; dihydrolipoamide dehydrogenase | 1.8.1.4 | K00382 | A04.441 |
| ENO, eno; enolase | 4.2.1.11 | K01689 | A01.1414 |
| FBA, fbaA; fructose-bisphosphate aldolase, class II | 4.1.2.13 | K01624 | A09.246 |
| FBA, fbaA; fructose-bisphosphate aldolase, class II | 4.1.2.13 | K01624 | A12.676 |
| FBP, fbp; fructose-1,6-bisphosphatase I | 3.1.3.11 | K03841 | A02.911 |
| frmA, ADH5, adhC; S-(hydroxymethyl)glutathione dehydrogenase / alcohol dehydrogenase | 1.1.1.1 | K00121 | A06.941 |
| galM, GALM; aldose 1-epimerase | 5.1.3.3 | K01785 | A02.1088 |
| galM, GALM; aldose 1-epimerase | 5.1.3.3 | K01785 | A06.1271 |
| galM, GALM; aldose 1-epimerase | 5.1.3.3 | K01785 | A06.1272 |
| galM, GALM; aldose 1-epimerase | 5.1.3.3 | K01785 | A08.52 |
| GAPDH, gapA; glyceraldehyde 3-phosphate dehydrogenase | 1.2.1.12 | K00134 | A04.471 |
| Glucose-6-phosphate 1-epimerase | 5.1.3.15 | K01792 | A01.712 |
| GPI, pgi; glucose-6-phosphate isomerase | 5.3.1.9 | K01810 | A11.269 |
| gpmI; 2,3-bisphosphoglycerate-independent phosphoglycerate mutase | 5.4.2.12 | K15633 | A02.390 |
| HK; hexokinase | 2.7.1.1 | K00844 | A03.1024 |
| HK; hexokinase | 2.7.1.1 | K00844 | A06.712 |
| pckA; phosphoenolpyruvate carboxykinase (ATP) | 4.1.1.49 | K01610 | A07.224 |
| PDC, pdc; pyruvate decarboxylase | 4.1.1.1 | K01568 | A09.602 |
| PDHA, pdhA; pyruvate dehydrogenase E1 component *α* subunit | 1.2.4.1 | K00161 | A02.1011 |
| PDHB, pdhB; pyruvate dehydrogenase E1 component *β* subunit | 1.2.4.1 | K00162 | A06.232 |
| pfkA, PFK; 6-phosphofructokinase 1 | 2.7.1.11 | K00850 | A08.793 |
| PGK, pgk; phosphoglycerate kinase | 2.7.2.3 | K00927 | A02.415 |
| pgm; phosphoglucomutase | 5.4.2.2 | K01835 | A02.457 |
| PK, pyk; pyruvate kinase | 2.7.1.40 | K00873 | A01.1075 |
| TPI, tpiA; triosephosphate isomerase (TIM) | 5.3.1.1 | K01803 | A10.480 |

**Table S3 Putative genes involved in** **the pathway of terpenoid backbone biosynthesis in *Daedaleopsis sinensis*.**

| **Gene name and definition** | **Enzyme code** | **KO term** | **Gene ID** |
| --- | --- | --- | --- |
| atoB; acetyl-CoA C-acetyltransferase | 2.3.1.9 | K00626 | A06.782 |
| DHDDS, RER2, SRT1; ditrans,polycis-polyprenyl diphosphate synthase | 2.5.1.87 | K11778 | A02.906 |
| FDPS; farnesyl diphosphate synthase | 2.5.1.1 | K00787 | A01.319 |
| FDPS; farnesyl diphosphate synthase | 2.5.1.10 | K00787 | A01.319 |
| FNTA; protein farnesyltransferase / geranylgeranyltransferase type-1 subunit *α* | 2.5.1.58 | K05955 | A12.331 |
| FNTB; protein farnesyltransferase subunit *β* | 2.5.1.58 | K05954 | A11.688 |
| GGPS1; geranylgeranyl diphosphate synthase, type III | 2.5.1.1 | K00804 | A03.131 |
| GGPS1; geranylgeranyl diphosphate synthase, type III | 2.5.1.10 | K00804 | A03.131 |
| GGPS1; geranylgeranyl diphosphate synthase, type III | 2.5.1.29 | K00804 | A03.131 |
| HMGCR; hydroxymethylglutaryl-CoA reductase (NADPH) | 1.1.1.34 | K00021 | A10.683 |
| Hydroxymethylglutaryl-CoA synthase | 2.3.3.10 | K01641 | A06.1111 |
| ICMT, STE14; protein-S-isoprenylcysteine O-methyltransferase | 2.1.1.100 | K00587 | A06.256 |
| ICMT, STE14; protein-S-isoprenylcysteine O-methyltransferase | 2.1.1.100 | K00587 | A10.814 |
| IDI; isopentenyl-diphosphate *γ*-isomerase | 5.3.3.2 | K01823 | A01.739 |
| mvaK2; phosphomevalonate kinase | 2.7.4.2 | K00938 | A07.624 |
| MVD, mvaD; diphosphomevalonate decarboxylase | 4.1.1.33 | K01597 | A07.754 |
| NUS1; dehydrodolichyl diphosphate syntase complex subunit NUS1 | 2.5.1.87 | K19177 | A01.775 |
| PCYOX1, FCLY; prenylcysteine oxidase / farnesylcysteine lyase | 1.8.3.5 | K05906 | A10.110 |
| PCYOX1, FCLY; prenylcysteine oxidase / farnesylcysteine lyase | 1.8.3.6 | K05906 | A10.110 |
| RCE1, FACE2; prenyl protein peptidase | FACE2 | K08658 | A12.114 |
| STE24; STE24 endopeptidase | 3.424.84 | K06013 | A11.491 |

**Table S4 Putative genes involved in** **the pathway of pyruvate metabolism in *Daedaleopsis sinensis*.**

| **Gene name and definition** | **Enzyme code** | **KO term** | **Gene ID** |
| --- | --- | --- | --- |
| pckA; phosphoenolpyruvate carboxykinase (ATP) | 4.1.1.49 | K01610 | A07.224 |
| PK, pyk; pyruvate kinase | 2.7.1.40 | K00873 | A01.1075 |
| LDHD, dld; D-lactate dehydrogenase (cytochrome) | 1.1.2.4 | K00102 | A02.141 |
| LDHD, dld; D-lactate dehydrogenase (cytochrome) | 1.1.2.4 | K00102 | A09.870 |
| DLD3; (R)-2-hydroxyglutarate---pyruvate transhydrogenase | 1.1.99.40 | K21618 | A01.610 |
| GRHPR; glyoxylate/hydroxypyruvate reductase | 1.1.1.79 | K00049 | A10.682 |
| GLO1, gloA; lactoylglutathione lyase | 4.4.1.5 | K01759 | A07.1031 |
| gloB, gloC, HAGH; hydroxyacylglutathione hydrolase | 3.1.2.6 | K01069 | A02.592 |
| lldD; L-lactate dehydrogenase (cytochrome) | 1.1.2.3 | K00101 | A01.584 |
| lldD; L-lactate dehydrogenase (cytochrome) | 1.1.2.3 | K00101 | A03.350 |
| lldD; L-lactate dehydrogenase (cytochrome) | 1.1.2.3 | K00101 | A03.353 |
| E.1.13.12.4; lactate 2-monooxygenase | 1.13.12.4 | K00467 | A09.140 |
| ackA; acetate kinase | 2.7.2.1 | K00925 | A08.342 |
| ALDH; NAD^+^ | 1.2.1.3 | K00128 | A01.47 |
| ALDH; NAD^+^ | 1.2.1.3 | K00128 | A06.747 |
| ALDH7A1; aldehyde dehydrogenase family 7 member A1 | 1.2.1.3 | K14085 | A03.752 |
| ACSS, acs; acetyl-CoA synthetase | 6.2.1.1 | K01895 | A03.858 |
| leuA, IMS; 2-isopropylmalate synthase | 2.3.3.13 | K01649 | A08.676 |
| leuA, IMS; 2-isopropylmalate synthase | 2.3.3.13 | K01649 | A11.256 |
| LYS21, LYS20; homocitrate synthase | 2.3.3.14 | K01655 | A01.1337 |
| ACACA; acetyl-CoA carboxylase / biotin carboxylase 1 | 6.4.1.2 | K11262 | A05.392 |
| E.2.3.1.9, atoB; acetyl-CoA C-acetyltransferase | 2.3.1.9 | K00626 | A06.782 |
| PDHA, pdhA; pyruvate dehydrogenase E1 component *α* subunit | 1.2.4.1 | K00161 | A02.1011 |
| PDHB, pdhB; pyruvate dehydrogenase E1 component *β* subunit | 1.2.4.1 | K00162 | A06.232 |
| DLD, lpd, pdhD; dihydrolipoamide dehydrogenase | 1.8.1.4 | K00382 | A04.441 |
| DLAT, aceF, pdhC; pyruvate dehydrogenase E2 component (dihydrolipoamide acetyltransferase) | 2.3.1.12 | K00627 | A01.1006 |
| aceB, glcB; malate synthase | 2.3.3.9 | K01638 | A07.860 |
| E.4.2.1.2B, fumC, FH; fumarate hydratase, class II | 4.2.1.2 | K01679 | A01.575 |
| MDH2; malate dehydrogenase | 1.1.1.37 | K00026 | A02.420 |
| MDH2; malate dehydrogenase | 1.1.1.37 | K00026 | A06.1116 |
| E1.1.1.40, maeB; malate dehydrogenase (oxaloacetate-decarboxylating) (NADP^+^) | 1.1.1.40 | K00029 | A12.196 |
| ME2, sfcA, maeA; malate dehydrogenase (oxaloacetate-decarboxylating) | 1.1.1.38 | K00027 | A01.1452 |
| ME2, sfcA, maeA; malate dehydrogenase (oxaloacetate-decarboxylating) | 1.1.1.38 | K00027 | A01.1479 |
| PC, pyc; pyruvate carboxylase | 6.4.1.1 | K01958 | A05.460 |
| PC, pyc; pyruvate carboxylase | 6.4.1.1 | K01958 | A05.461 |

**Table S5 Putative genes involved in** **the pathway of phenylalanine metabolism in *Daedaleopsis sinensis*.**

| **Gene name and definition** | **Enzyme code** | **KO term** | **Gene ID** |
| --- | --- | --- | --- |
| PAL; phenylalanine ammonia-lyase | 4.3.1.24 | K10775 | A03.399 |
| GOT1; aspartate aminotransferase, cytoplasmic | 2.6.1.1 | K14454 | A02.872 |
| GOT2; aspartate aminotransferase, mitochondrial | 2.6.1.1 | K14455 | A01.665 |
| hisC; histidinol-phosphate aminotransferase | 2.6.1.9 | K00817 | A10.735 |
| ARO8; aromatic amino acid aminotransferase I / 2-aminoadipate transaminase | 2.6.1.5 | K00838 | A03.252 |
| ARO8; aromatic amino acid aminotransferase I / 2-aminoadipate transaminase | 2.6.1.5 | K00838 | A03.255 |
| ARO8; aromatic amino acid aminotransferase I / 2-aminoadipate transaminase | 2.6.1.5 | K00838 | A02.933 |
| ARO8; aromatic amino acid aminotransferase I / 2-aminoadipate transaminase | 2.6.1.5 | K00838 | A03.491 |
| ARO8; aromatic amino acid aminotransferase I / 2-aminoadipate transaminase | 2.6.1.5 | K00838 | A06.593 |
| ARO8; aromatic amino acid aminotransferase I / 2-aminoadipate transaminase | 2.6.1.57 | K00838 | A02.933 |
| ARO8; aromatic amino acid aminotransferase I / 2-aminoadipate transaminase | 2.6.1.57 | K00838 | A03.252 |
| ARO8; aromatic amino acid aminotransferase I / 2-aminoadipate transaminase | 2.6.1.57 | K00838 | A03.255 |
| ARO8; aromatic amino acid aminotransferase I / 2-aminoadipate transaminase | 2.6.1.57 | K00838 | A03.491 |
| ARO8; aromatic amino acid aminotransferase I / 2-aminoadipate transaminase | 2.6.1.57 | K00838 | A06.593 |
| DDC, TDC; aromatic-_L_-amino-acid / _L_-tryptophan decarboxylase | 4.1.1.28 | K01593 | A03.279 |
| AOC3, AOC2, tynA; primary-amine oxidase | 1.4.3.21 | K00276 | A11.584 |
| AOC3, AOC2, tynA; primary-amine oxidase | 1.4.3.21 | K00276 | A10.570 |
| AOC3, AOC2, tynA; primary-amine oxidase | 1.4.3.21 | K00276 | A10.673 |
| MIF; phenylpyruvate tautomerase | 5.3.2.1 | K07253 | A02.244 |
| E.1.2.1.5; aldehyde dehydrogenase (NAD(P)+) | 1.2.1.5 | K00129 | A10.768 |
| amiE; amidase | 3.5.1.4 | K01426 | A03.902 |
| amiE; amidase | 3.5.1.4 | K01426 | A01.685 |
| amiE; amidase | 3.5.1.4 | K01426 | A01.235 |
| amiE; amidase | 3.5.1.4 | K01426 | A01.1174 |
| amiE; amidase | 3.5.1.4 | K01426 | A03.892 |
| amiE; amidase | 3.5.1.4 | K01426 | A01.690 |
| paaH, hbd, fadB, mmgB; 3-hydroxybutyryl-CoA dehydrogenase | 1.1.1.157 | K00074 | A05.49 |
| paaH, hbd, fadB, mmgB; 3-hydroxybutyryl-CoA dehydrogenase | 1.1.1.157 | K00074 | A03.133 |

**Table S6 Putative CAZy acting on fungal, plant, and bacterial cell walls in *Daedaleopsis sinensis* and *D. nitida*.**

| **CAZy acting component** | | **CAZy family** | ***Daedaleopsis sinensis*** | ***Daedaleopsis nitida*** |
| --- | --- | --- | --- | --- |
| Polysaccharides from plant cell wall | Cellulose | AA3 | 29 | 32 |
|  |  | AA8 | 1 | 2 |
|  |  | AA9 | 19 | 17 |
|  |  | GH1 | 2 | 2 |
|  |  | GH3 | 8 | 8 |
|  |  | GH5 | 22 | 23 |
|  |  | GH6 | 1 | 1 |
|  |  | GH7 | 4 | 2 |
|  |  | GH9 | 1 | 1 |
|  |  | GH12 | 4 | 4 |
|  |  | GH45 | 0 | 1 |
|  |  | GH51 | 2 | 2 |
|  |  | GH74 | 1 | 1 |
|  |  | GT2 | 20 | 16 |
|  |  | Total | 114 | 112 |
|  | Hemicellulase | CE1 | 2 | 2 |
|  |  | CE4 | 7 | 8 |
|  |  | CE12 | 3 | 3 |
|  |  | GH2 | 5 | 4 |
|  |  | GH3 | 8 | 8 |
|  |  | GH5 | 22 | 23 |
|  |  | GH10 | 4 | 5 |
|  |  | GH12 | 4 | 4 |
|  |  | GH16 | 30 | 32 |
|  |  | GH27 | 4 | 4 |
|  |  | GH30 | 2 | 2 |
|  |  | GH31 | 6 | 7 |
|  |  | GH35 | 4 | 4 |
|  |  | GH43 | 10 | 11 |
|  |  | GH51 | 2 | 2 |
|  |  | GH74 | 1 | 1 |
|  |  | GH93 | 1 | 1 |
|  |  | GH95 | 1 | 1 |
|  |  | GH115 | 1 | 1 |
|  |  | GT8 | 8 | 8 |
|  |  | Total | 125 | 131 |
|  | Pectin | CE1 | 2 | 2 |
|  |  | CE8 | 2 | 2 |
|  |  | CE12 | 3 | 3 |
|  |  | GH2 | 5 | 4 |
|  |  | GH3 | 8 | 8 |
|  |  | GH28 | 11 | 10 |
|  |  | GH35 | 4 | 4 |
|  |  | GH43 | 10 | 11 |
|  |  | GH51 | 2 | 2 |
|  |  | GH78 | 3 | 3 |
|  |  | GH88 | 1 | 1 |
|  |  | GH93 | 1 | 1 |
|  |  | GH105 | 4 | 5 |
|  |  | PL4 | 2 | 3 |
|  |  | Total | 58 | 59 |
| Fungal cell wall | *β*-Glucans | GH1 | 2 | 2 |
|  |  | GH3 | 8 | 8 |
|  |  | GH5 | 22 | 23 |
|  |  | GH7 | 4 | 2 |
|  |  | GH9 | 1 | 1 |
|  |  | GH12 | 4 | 4 |
|  |  | GH16 | 30 | 32 |
|  |  | GH17 | 1 | 1 |
|  |  | GH30 | 2 | 2 |
|  |  | GH72 | 1 | 1 |
|  |  | GT48 | 2 | 2 |
|  |  | Total | 77 | 78 |
|  | Chitin | GT2 | 20 | 16 |
|  |  | CE4 | 7 | 8 |
|  |  | GH18 | 21 | 19 |
|  |  | GH20 | 6 | 6 |
|  |  | Total | 54 | 49 |
|  | *α*-Glucans and other FCWP | GH13 | 9 | 10 |
|  |  | GH71 | 5 | 5 |
|  |  | GH79 | 12 | 14 |
|  |  | Total | 26 | 29 |
| Non-plant polysaccharides | Bacterial or animal polysaccharides | PL8 | 4 | 4 |
|  |  | PL14 | 7 | 6 |
|  |  | GH79 | 12 | 14 |
|  |  | GH88 | 1 | 1 |
|  |  | Total | 24 | 25 |
| Basal carbohydrate metabolism | Free carbohydrates | GH13 | 9 | 10 |
|  |  | GH15 | 2 | 3 |
|  |  | GH37 | 2 | 2 |
|  |  | GT3 | 1 | 1 |
|  |  | GT4 | 4 | 4 |
|  |  | GT20 | 3 | 3 |
|  |  | GT35 | 1 | 1 |
|  |  | Total | 22 | 24 |


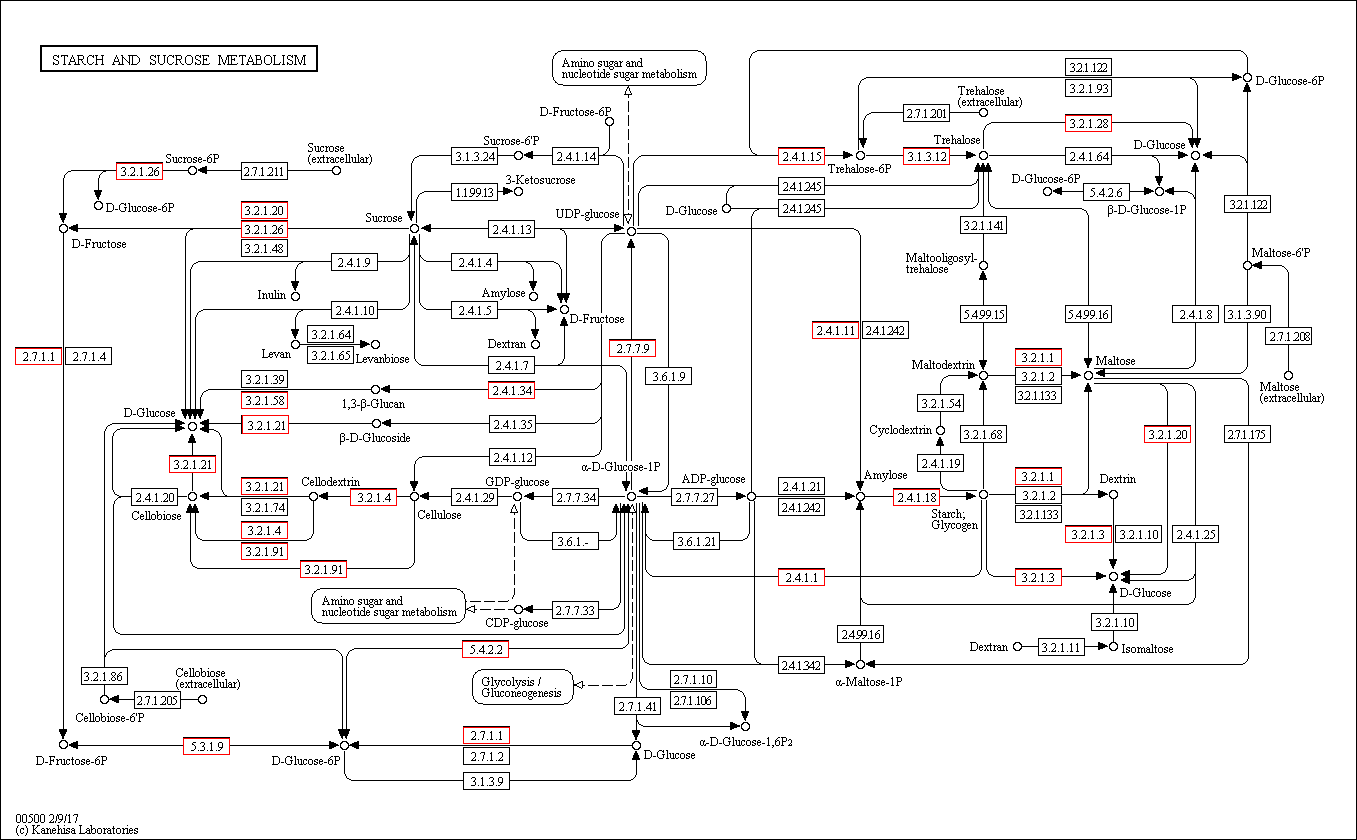


**FIGURE S1 Starch and sucrose metabolic pathways in Daedaleopsis sinensis.**


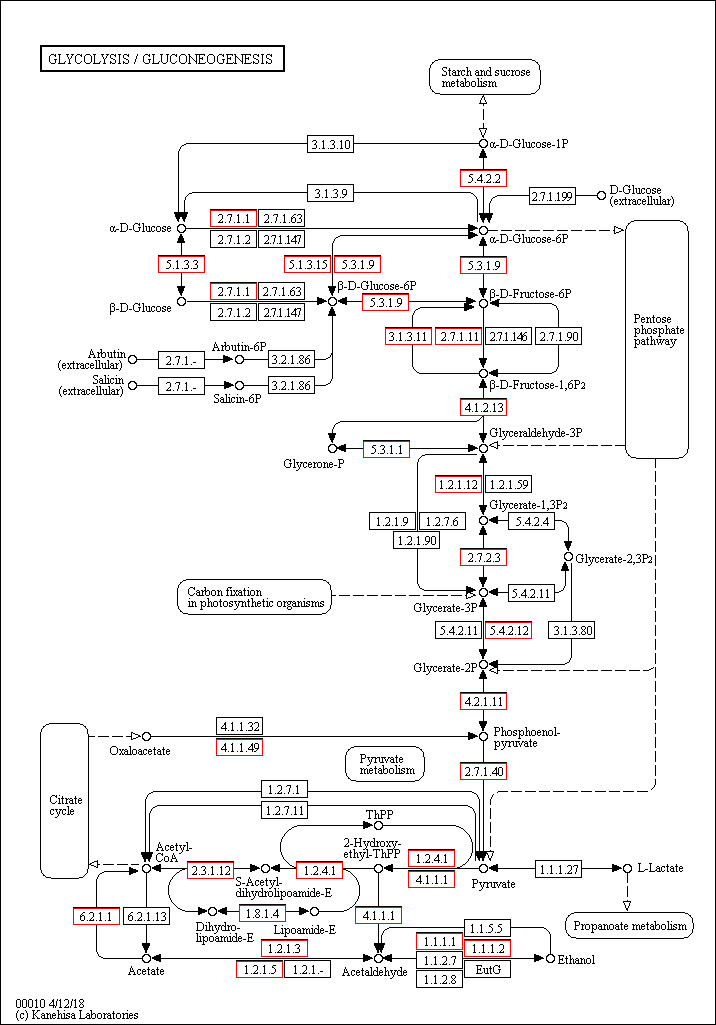


**FIGURE S2 Glycolysis / gluconeogenesis pathways in *Daedaleopsis sinensis.***


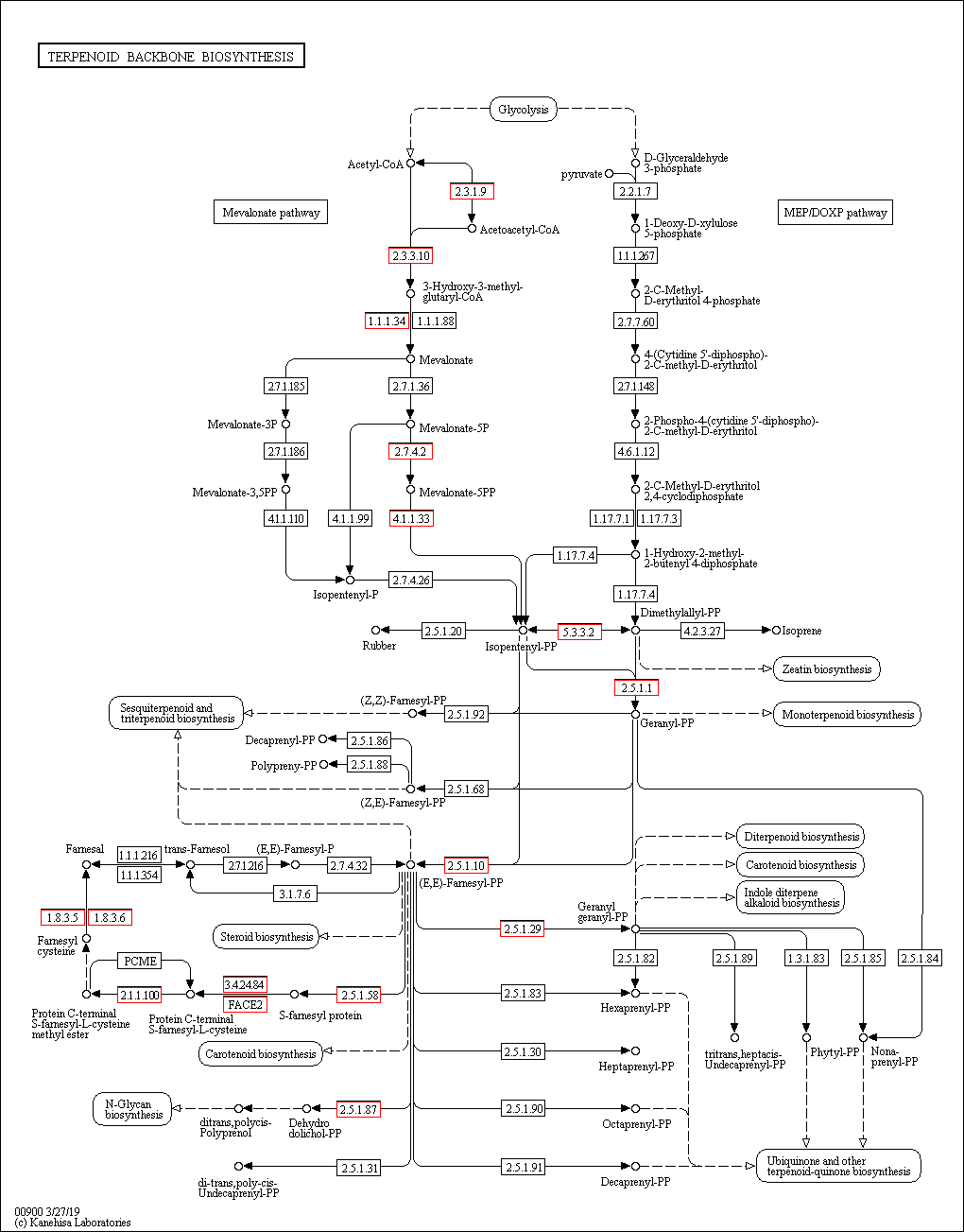


**FIGURE S3 Terpenoid backbone biosynthesis pathways in *Daedaleopsis sinensis*.**


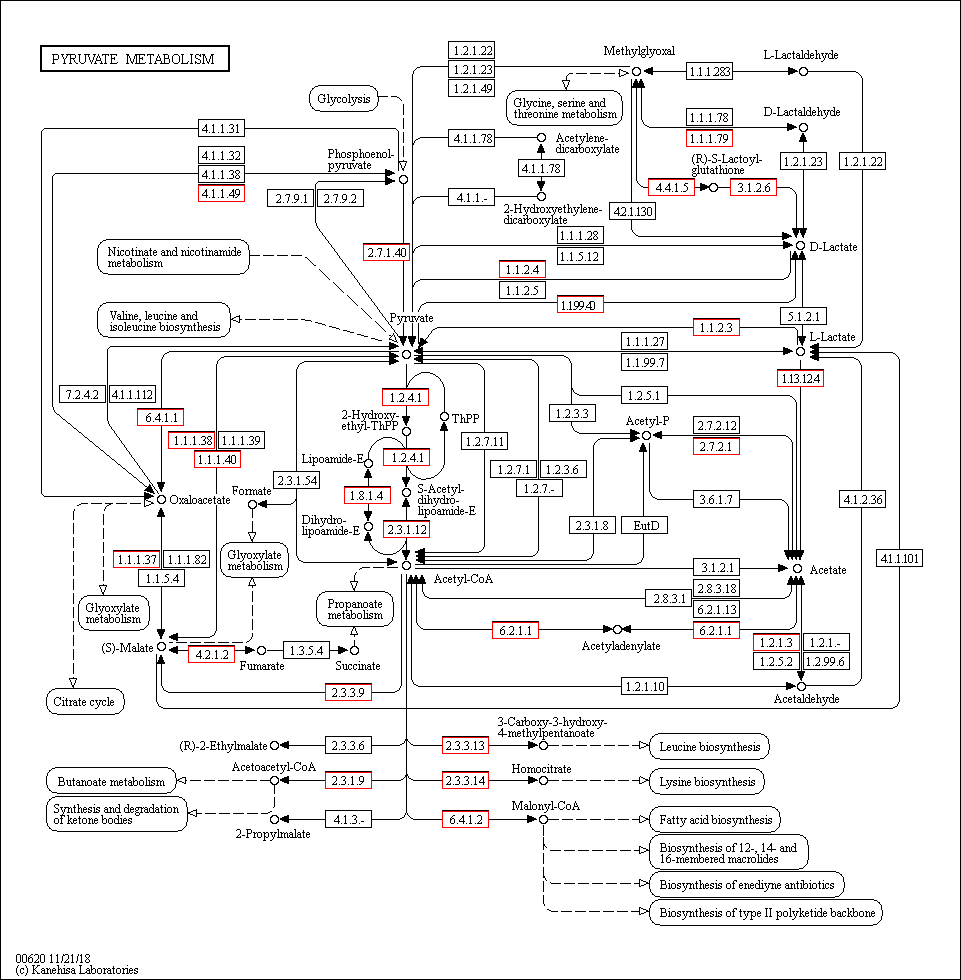


**FIGURE S4 Pyruvate metabolic pathways in *Daedaleopsis sinensis*.**


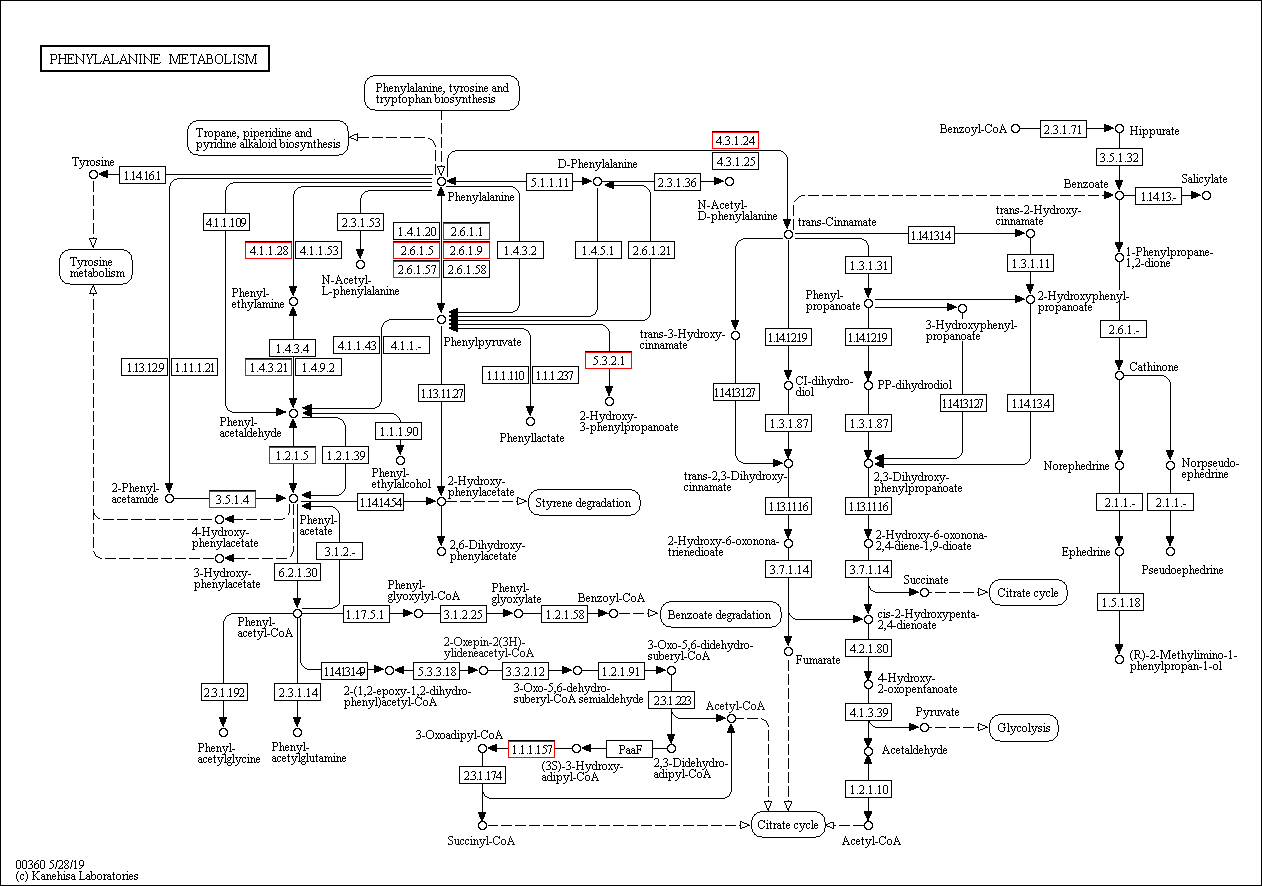


**FIGURE S5 Phenylalanine metabolic pathways in *Daedaleopsis sinensis*.**
